# Sex-Specific patterns of vulnerability to alcohol addiction-like behaviors in rats

**DOI:** 10.1038/s41398-026-03825-w

**Published:** 2026-01-29

**Authors:** Anna Maria Borruto, Andrea Coppola, Leon Höglund, Sandra Eriksson Solander, Michele Petrella, Markus Heilig, Eric Augier

**Affiliations:** Center for Social and Affective Neuroscience, Department of Biomedical and Clinical Sciences, Linköping University, S-581 85 Linköping, Sweden

**Keywords:** Addiction, Neuroscience

## Abstract

Only a minority of alcohol users develop alcohol use disorder (AUD), and the extent to which vulnerability to this condition depends on sex remains insufficiently explored in preclinical research. Using an established model that reverse-translates key diagnostic criteria for AUD, we investigated this question in male and female rats. Criteria for addiction-like behavior assessed were: (i) the inability to refrain from alcohol-seeking, (ii) high motivation for alcohol, and (iii) continued alcohol use despite negative consequences, assessed using footshock punishment. We found that a larger proportion of females (12.90%) met all three criteria compared to males (6.45%). Sex-differences observed were independent of alcohol consumption history, footshock sensitivity, or basal anxiety levels. Factor analysis results support the existence of both shared and sex-specific behavioral dimensions underlying addiction vulnerability. Notably, while persistence in alcohol-seeking and motivation loaded similarly onto “Factor 1” in both sexes, resistance to punishment showed opposite loadings on “Factor 3” in males and females. Moreover, this factor was differentially correlated with the global addiction score across sexes, indicating that this behavioral dimension may contribute differently to addiction-like behaviors in males and females. Notably, impulsivity was strongly correlated with the number of addiction-like criteria in both male and female rats, underscoring its broad role in shaping the risk. In contrast, neither anxiety-like behavior, locomotor activity in a novel environment, nor social dominance were predictors of addiction-like behaviors. These results emphasize the need for sex-specific approaches in AUD research, revealing complex behavioral traits that influence addiction risk.

## Introduction

Alcohol Use Disorder (AUD) is one of the leading causes of disease and premature mortality worldwide [1, 2]. Despite its detrimental consequences, AUD remains among the most undertreated mental health disorders, with limited progress in treatment efficacy over recent decades [3]. People with AUD often exhibit compulsive patterns of alcohol consumption, characterized by loss of control and continued use despite severe personal, social, and health consequences [4, 5]. These behaviors are driven by complex neurobiological and psychosocial mechanisms, many of which remain poorly understood, particularly regarding individual variability and sex-specific factors. This gap highlights the urgent need for research aimed at developing more effective and targeted interventions.

A major factor contributing to the high prevalence of AUD compared to other substance use disorder (SUD) is the easy accessibility of alcohol and its widespread recreational use [6]. However, not all users develop addiction [7–9], and individual risk is shaped by a complex interplay of genetic, neurobiological, environmental, social, and personality factors [10–12]. No single factor fully explains individual vulnerability to AUD. Among risk factors, externalizing traits, such as impulsivity and risk-taking, are associated with higher vulnerability for AUD [13–15]. However, internalizing traits like neuroticism also show an association with AUD risk [15, 16] and the high comorbidity of AUD with anxiety disorders [17–20] suggests that alcohol may be used to cope with negative emotional states, which would promote alcohol use and addiction risk through negative reinforcement [21].

Furthermore, social stressors, such as peer pressure, and socioeconomic factors like education and income, also influence sensitivity to the reinforcing properties of drugs of abuse [22–25]. Sex also plays a crucial role in the development of AUD, with notable differences between men and women in the reasons for initiating alcohol use and the addiction trajectory. Men are more likely to drink and relapse due to social factors and positive emotions, driven by peer acceptance, especially in adolescence [2, 26–28]. In contrast, women often use alcohol to cope with negative emotions like anxiety, depression, and stress, increasing relapse risk [29–32]. These differences underscore the need for sex-specific approaches in AUD prevention and treatment. However, most preclinical studies on AUD have focused on males, limiting our understanding of sex-based differences [33–35].

Individual differences also play a crucial role in preclinical models [36–38]. Recent studies suggest that only a subset of rodents develop addiction-like behaviors after prolonged drug exposure. Indeed, while all rodents trained in operant alcohol self-administration consume comparable amounts of alcohol rewards [39, 40], only some exhibit addiction-like behavior, such as aversion-resistant alcohol seeking and taking [41, 42] or choice of alcohol over natural rewards [43, 44]. To investigate individual differences in vulnerability for addiction-like behaviors, a preclinical multi-symptom model was developed [45]. This model is based on behaviors thought to reflect clinical manifestations of AUD, as described in the Diagnostic and Statistical Manual of Mental Disorders (DSM-5). It classifies experimental animals based on three key addiction-like behaviors: (i) inability to withhold drug-seeking when drug is absent (persistence of alcohol seeking) [46], heightened motivation to obtain the drug (motivation), and (iii) persistent drug-seeking despite adverse consequences (resistance to punishment). Originally developed for cocaine addiction [45, 47, 48], this multi-symptom model was recently adapted for alcohol, providing a framework for exploring individual vulnerability to AUD [49–51]. However, similar to many preclinical models, studies using this model for both cocaine and alcohol have largely excluded females, limiting insights into sex-specific variability and leaving a significant gap in the understanding of AUD mechanisms in women.

In this study, we aimed to address these gaps by employing this multi-symptom model to investigate individual differences in AUD in both male and female Wistar rats. Furthermore, we explored the role of anxiety-like behaviors and social hierarchy as potential predictors of vulnerability to AUD. By integrating these factors, our study seeks to provide a comprehensive understanding of how individual traits and social dynamics interact to influence AUD vulnerability in a sex-specific manner.

## Materials and methods

A detailed description of the materials and methods is available in the Supplementary Materials.

### Animals

**Fig. 1:**
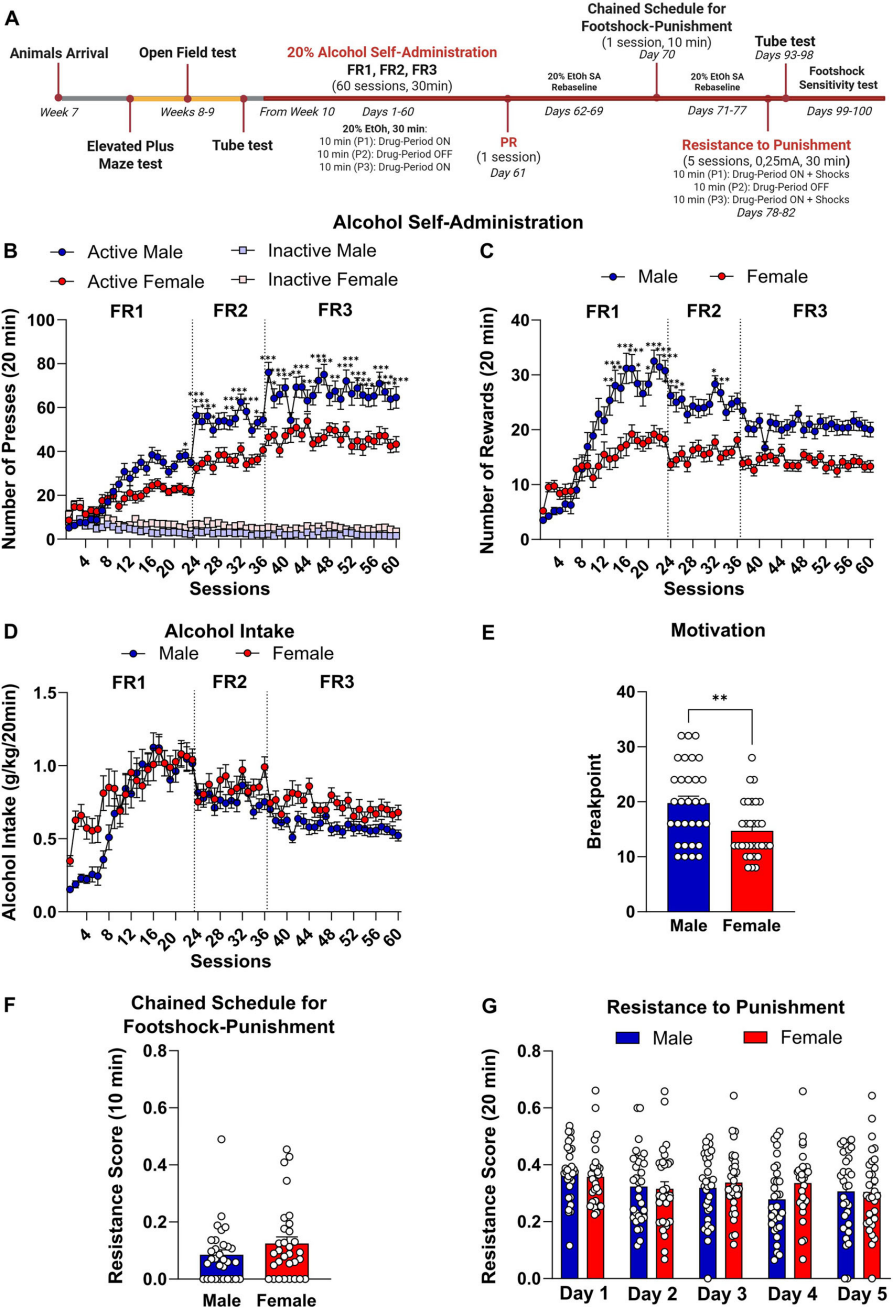
Sex Differences in Alcohol Self-Administration, Motivation and Resistance to Punishment in Male and Female Rats. **A** Schematic representation of the experimental timeline illustrating the behavioral procedures. **B** Number of active (circle) and inactive (square) lever presses over 60 days of 20% alcohol self-administration under FR1 (sessions 1–23), FR2 (sessions 24–36), and FR3 (sessions 37–60) schedules of reinforcement in male and female Wistar rats (n = 31/sex). **C** Total number of alcohol rewards earned over 60 days of self-administration by male and female rats. **D** Alcohol intake (g/kg), calculated as the number of rewards normalized by body weight, during alcohol self-administration training. **E** Breakpoints achieved during a progressive ratio session for 20% alcohol. **F** Resistance scores recorded during a single 10 min foot-shock session (0.25 mA) under a chained FR3 schedule of reinforcement. **G** Resistance scores recorded during five punished sessions using a pseudorandom punishment schedule of reinforcement (0.25 mA, FR3). Values are presented as mean ± SEM. *p < 0.05, **p < 0.01, ***p < 0.001 indicate significant sex differences (male vs. female).

Male (n = 32) and female (n = 32) Wistar rats (Charles River, Germany) were used in this study. At the start of the experiment, 8-week-old rats weighed 270–300 g (males) and 190–220 g (females). They were housed in groups of four in individually ventilated cages from the animal facility at Linköping University. The rats were maintained in a specific pathogen free (SPF) environment under a 12 h reversed light/dark cycle. Animal care and experimental procedures were carried out in accordance with the European Union Directive 2010/63/EU and Swedish laws. The protocol was approved by the Animal Ethics Committee (Jordbruksverket, Dnr 01680-2020 ID1942). The experimental design is summarized in Fig. 1A.

### Elevated plus maze test

To assess basal anxiety-like behavior, animals underwent the elevated plus maze test, where spontaneous exploration of open versus enclosed spaces served as the primary measure [52].

### Open field test

To evaluate basal exploratory behavior and locomotory reactivity to a novel environment, the animals underwent an open field test as described previously [43].

### Tube test

To determine social rank, we used the confrontation tube test, in which the performance of the animals in pushing a cage mate out of a plexiglass tube serves as a proxy of social dominance [53]. Each animal’s rank score was based on the number of confrontations won, and rats were ranked from highest to lowest (α > β > γ > ω). The top two were classified as dominant, and the bottom two as subordinate.

### Operant alcohol self-administration

Operant- and drug-naïve rats were trained to self-administer 20% (v/v; Solveco, Roserberg, Sweden) alcohol without prior sucrose or saccharin fading [40, 54].

### Evaluation of the three criteria for alcohol-related behaviors

Rats underwent 60 alcohol self-administration sessions (30 min/day, 5–6 days/week). Each session included two 10-min drug periods (start and end) and a 10-min no-drug period (middle). During drug periods, each active lever press delivered 0.1 ml alcohol and a 5-sec cue light, followed by a 10-sec time-out where lever responses had no effect but were recorded as a measure of impulsivity [55]. In the no-drug period (house light on), active lever presses were not rewarded. Inactive lever responses were always recorded but had no effect. Training began on a fixed-ratio 1 (FR1) schedule, progressing to FR2 (sessions 24–36) and FR3 (sessions 37–60). Two rats (1 female, 1 male) were excluded for failing to acquire the behavior ( ≤ 3 active presses). A summary of behavioral measures and session windows used to define each addiction-like criterion is reported in Table S1.

### Persistence in alcohol-seeking

To measure persistence in alcohol-seeking behavior, we recorded the number of responses on the active lever during the no-drug periods across all training sessions. For the evaluation of addiction-like criteria, the average number of responses from the FR3 training sessions (sessions 37–60) was used to minimize behavioral fluctuations [51].

### Motivation

To assess the motivation to seek alcohol, rats were tested under a progressive ratio (PR) schedule of reinforcement [56], where the required number of active lever presses increased progressively following the formula: 1, 2, 3, 4, 6, 8, 10, 12, 16, 20, 24, 28, 32, 36, etc. A 0.1 ml alcohol reward was delivered once the ratio was met, followed by the illumination of the cue light for 5 s and a 5 s time-out. The session ended after 30 min without reinforcement. The highest ratio completed, referred to as the breakpoint, was used for the final evaluation of addiction-like criteria.

### Resistance to punishment

To assess continued alcohol use despite negative consequences, rats underwent punished self-administration sessions involving footshock. We first used a protocol previously employed in the multi-symptom model [49, 51], where during a 10 min chained schedule, three active lever presses (FR3) triggered a sequence: the first illuminated a green cue, the second delivered a 0.25 mA footshock (0.5 s), and the third delivered 20% alcohol. If the sequence was not completed within 1 min, all cues were terminated, and the sequence restarted. Since a single 10 min session may not adequately reveal individual differences in punishment-resistant responding [57, 58], a second approach involving multiple sessions was used. Adapting a previously established protocol [59, 60], rats received a 0.25 mA footshock (0.5 s) every 8th active press during 10 min drug periods, punishing only one in three reward cycles and decoupling punishment from alcohol delivery. Animals underwent five punished sessions. Resistance scores were calculated as the ratio of punished alcohol deliveries to the sum of punished and mean unpunished deliveries (from the last three non-punished sessions). The average resistance score from these five punished sessions was used to classify the animals within the multi-symptom model.

### Evaluation of addict-like and non-addict-like rats

A rat was considered positive for a specific addiction-like criterion if its individual score for each addiction-like behavior exceeded the 66th percentile of the total distribution, in line with previous studies on alcohol addiction using multi-symptom model [49, 51]. The analysis was conducted separately for males and females, treating the sexes as independent populations, to specifically assess within-sex vulnerability. Animals were categorized based on whether they were positive for 0, 1, 2, or 3 criteria. Finally, a Global Addiction Score (GAS) was calculated for each subject as the sum of the normalized z-scores for all criteria [61]. Off note, we performed a principal component analysis (PCA) including all addiction-like behavioral measures in male and female rats, considering sex as a factor (Fig. S1). Notably, animals with a higher number of positive addiction-like criteria were consistently located toward the same regions of the PCA space, confirming that the 66th percentile–based classification aligns with the overall behavioral structure revealed by this unbiased multivariate analysis.

### Footshock sensitivity test

Animals were placed in the operant chamber, and footshock (0.5 s) was delivered starting from 0.05 mA, with the intensity increasing by 0.05 mA every 30 s [43, 62].

### Data analysis

The optimum sample sizes and animal numbers were determined by power analysis of pre-existing literature [49, 51]. Laboratory animals were evaluated in a blind manner throughout all phases of the experiments and were randomly assigned to experimental groups based on three criteria they met. Before conducting analyses of variance (ANOVA) or parametric tests, we ensured that the assumptions of homogeneity of variance and normality were met. Homogeneity of variance was assessed with Levene’s test, while the Shapiro-Wilk test was used to evaluate the normality of data distribution. When two or more independent variables were considered, data were analyzed using two- or three-way ANOVA, followed by Tukey’s post-hoc test when appropriate. One-way ANOVA was used for comparisons among three or more groups. When normality assumptions were not met, the Kruskal-Wallis test was applied, followed by Dunn’s post-hoc test. Comparisons between two groups were performed using unpaired t-tests, or Mann–Whitney tests in case of non-normal distributions. Pearson’s correlation analysis was used to assess associations between variables. Factor analysis was performed using principal component extraction followed by Varimax rotation. Data are expressed as mean ± SEM, and statistical significance was set at *p* < 0.05.

## Results

### Distinct patterns of alcohol self-administration, motivation and resistance to punishment in male and female rats

We first evaluated sex differences in alcohol-related behaviors using an established alcohol self-administration protocol [49, 51]. Male rats demonstrated higher lever-pressing rates for alcohol than females, with both sexes clearly differentiating between the active and inactive levers [sex: F_(1, 60)_ = 13.53, *p* < 0.001; time: F_(59, 3540)_ = 57.25, *p* < 0.001; time x sex: F_(59, 3540)_ = 6.42, *p* < 0.001; lever: F_(1, 60)_ = 517.44, p < 0.001; lever × sex: F_(59, 3540)_ = 100.29, *p* < 0.001; time × lever sex: F_(59, 3540)_ = 5.72, *p* < 0.001; Fig. 1B]. Total active lever presses are presented in fig. S2. Similarly, male rats earned a greater number of alcohol rewards compared to females [sex: F_(1, 60)_ = 23.07, *p* < 0.001; time: F_(59, 3540)_ = 29.48, *p* < 0.001; time × sex: F_(59, 3540)_ = 7.16, *p* < 0.001; Fig. 1C]. However, when alcohol intake was normalized by body weight, no significant differences were observed between the sexes [sex: F_(1, 60)_ = 3.71, *p* > 0.05; time: F_(59, 3540)_ = 22.54, *p* < 0.001; time × sex: F_(59, 3540)_ = 2.76, *p* < 0.001; Fig. 1D].

To evaluate impulsive behavior, we measured active lever pressing during time-out periods [55]. Male rats exhibited higher impulsive-like behavior, as indicated by a greater number of active lever responses compared to females fig. S3A). Additionally, while active lever pressing decreased from the initial sessions [21–23] to the late phase of training [58–60] in both sexes, male rats consistently displayed higher levels of responding than females fig. S3B).

Twenty-four hours after the last alcohol self-administration session, rats were tested using a progressive ratio schedule of reinforcement. Male rats displayed higher motivation to self-administer alcohol, as evidenced by higher breakpoints compared to females (U = 285.5, *p* < 0.01; Fig. 1E).

Next, we evaluated both sexes during a single punishment session using a chained schedule, as previously described [49]. No significant sex differences were observed during this session (U = 400.5, *p* > 0.05; Fig. 1F). Similarly, the intermittent punishment protocol also did not reveal any significant sex differences in resistance scores across sessions [sex: F_(1, 60)_ = 0.68, *p* > 0.05; time: F_(4, 240)_ = 5.50, *p* < 0.001; time × sex: F_(4, 240)_ = 1.98, *p* > 0.5; Fig. 1G].

### Alcohol addiction-like behaviors emerge in a subset of male and female rats

**Fig. 2:**
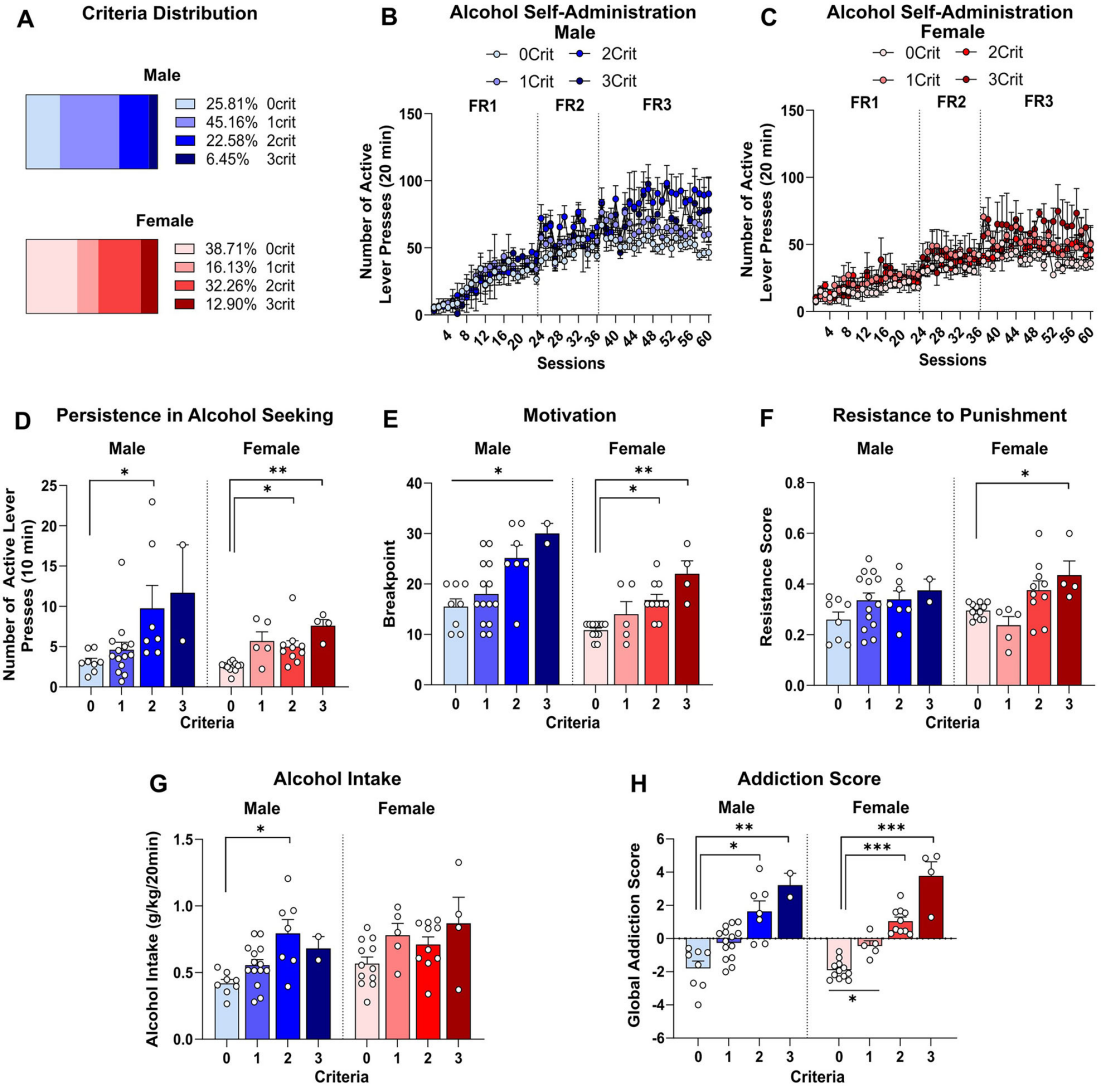
Only a Subset of Male and Female Rats Display Addiction-Like Behaviors. **A** Total distribution of animals based on the number of criteria met (0–3crit). Male rats: 0crit (25.81%), 1crit (45.16%), 2crit (22.58%), and 3crit (6.45%); female rats: 0crit (38.71%), 1crit (16.13%), 2crit (32.26%), and 3crit (12.90%). **B-C** Number of active lever presses during 60 days of 20% alcohol self-administration under FR1 (sessions 1–23), FR2 (sessions 24–36), and FR3 (sessions 37–60) schedules of reinforcement, grouped by the number of criteria met (0–3crit), for male and female Wistar rats (n = 31/sex). **D** Active lever presses recorded during the 10 min no-drug period. **E** Breakpoints achieved during a progressive ratio session for 20% alcohol. **F** Resistance scores recorded during five punished sessions using a pseudorandom punishment schedule of reinforcement (0.25 mA, FR3). **G** Alcohol intake (g/kg), calculated as the number of rewards normalized by body weight during alcohol self-administration training. **H** Global addiction score, calculated as the sum of normalized z-scores assigned to each criterion. The score was negative for the 0–1crit groups and positive for the 2–3crit groups in both sexes. Values are presented as mean ± SEM. *p < 0.05, **p < 0.01, ***p < 0.001 indicate significant criteria differences (0crit vs. 1crit vs. 2crit vs. 3crit).

After completion of the experiments, rats were retrospectively categorized based on the three criteria modeling clinical signs of loss of control over alcohol drinking. The animals were assigned to four groups (from 0crit to 3crit) based on the number of criteria met. In males, the majority were in the 0crit (25.81%) and 1crit (45.16%) groups, with fewer in the 2crit (22.58%) and 3crit (6.45%) groups. Females showed a different distribution, with a larger proportion in the 0crit (38.71%) and 2crit (32.26%) groups, followed by 1crit (16.13%) and 3crit (12.90%) groups (Fig. 2A).

No significant differences in active lever presses were observed between the groups meeting different criteria counts across the last three days of training (FR1: 22–24; FR2: 34–36; FR3: 58–60), neither for males [criteria: F_(3, 54)_ = 0.166, *p* = 0.919; time: F_(1, 54)_ = 0.160, *p* > 0.05; criteria × time: F_(3, 54)_ = 0.722, *p* > 0.05; Fig. 2B] nor females [criteria: F_(3, 54)_ = 0.908, *p* > 0.05; time: F_(1, 54)_ = 7.154, *p* < 0.05; criteria × time: F_(3, 54)_ = 0.847, *p* > 0.05; Fig. 2C].

When we assessed active lever pressing during the 10 s timeout, we observed that male rats in the 0crit and 1crit groups reduced active lever pressing during the training sessions, while the 2crit and 3crit groups maintained stable responses, indicating more impulsive-like behavior in the latter fig. S3C). Females showed a similar but less pronounced pattern fig. S3D).

Furthermore, during the no-drug period, significant group differences in alcohol-seeking behavior emerged in both males (H = 12.23, *p* < 0.01; Fig. 2D) and females (H = 17.36, *p* < 0.001; Fig. 2D). Alcohol-seeking rates increased progressively across groups, from 0crit to 3crit, in males (0crit: 3.11 ± 1.28; 1crit: 4.60 ± 3.55; 2crit: 9.76 ± 7.47; 3crit: 11.67 ± 8.43) and females (0crit: 2.52 ± 0.59; 1crit: 5.69 ± 2.59; 2crit: 5.00 ± 2.36; 3crit: 7.59 ± 1.57).

During the progressive ratio schedule, males showed a significant group effect (H = 11.15, *p* < 0.05; Fig. 2E), with 3crit (30.00 ± 2.83) rats displaying higher breakpoint values compared to 0crit (15.50 ± 4.38), 1crit (18.00 ± 6.18) and 2crit (25.14 ± 6.82) rats. Females exhibited a similar trend (H = 16.55, *p* < 0.001; Fig. 2E), with 3crit (22.0 ± 5.16) rats showing higher responses than 0crit (10.83 ± 1.59) and 1crit (14.00 ± 5.66) rats, but not differing from 2crit (16.80 ± 3.68) rats.

In the resistance to punishment, males showed no significant differences between groups (H = 3.917, *p* > 0.05; 0crit: 0.26 ± 0.08; 1crit: 0.34 ± 0.11; 2crit: 0.34 ± 0.09; 3crit: 0.38 ± 0.06; Fig. 2F), while females demonstrated greater resistance score in the 3crit (0.44 ± 0.11) group compared to the 0crit (0.30 ± 0.03) group [F_(3, 27)_ = 5.836, *p* < 0.01; Fig. 2F]. Across the five punished alcohol sessions, a main effect of the criteria was observed only in females fig. S4B), and not in males fig. S4A). In addition, neither male nor female rats showed differences in footshock sensitivity between groups classified based on criteria count, as assessed by the footshock sensitivity test (males: H = 4.393, *p* > 0.05; females: H = 2.625, *p* > 0.05; fig. S6B).

We also examined the relationship between alcohol intake (g/kg) and addiction-like behaviors. In males, alcohol consumption differed significantly between groups (H = 11.52, *p* < 0.01; Fig. 2G), with 2crit (0.73 ± 0.28) rats drinking more than 0crit (0.38 ± 0.09) and 1crit (0.66 ± 0.11) rats, though no differences were observed between 3crit (0.68 ± 0.12) and 0crit groups. In females, no significant differences were found between criteria groups [F_(3, 27)_ = 3.559, *p* = 0.0759; 0crit: 0.65 ± 0.17; 1crit: 0.73 ± 0.18; 2crit: 0.68 ± 0.13; 3crit: 0.87 ± 0.39; Fig. 2G]. A similar pattern was observed in the number of rewards. Male rats showed differences between the 0crit and 2crit groups fig. S6A). In females, the 3crit group earned significantly more rewards that the 0crit group fig. S6A).

Finally, we calculated the addiction score for each rat by summing the normalized z-scores assigned to each criterion. In males, the addiction score was negative for the 0–1crit groups (0crit: −1.79 ± 1.24; 1crit: −0.25 ± 0.98) and positive for the 2–3crit groups (2crit: 1.46 ± 1.76; 3crit: 3.22 ± 1.03; H = 17.94, *p* < 0.001; Fig. 2H). Females showed a similar trend, with negative scores for 0–1crit groups (0crit: −1.91 ± 0.55; 1crit: −0.44 ± 0.68) and positive for 2–3crit groups [2crit: 1.05 ± 0.78; 3crit: 3.77 ± 1.71; F_(3, 27)_ = 52.11, *p* < 0.001; Fig. 2H].

### Anxiety-like behavior and social hierarchy do not predict alcohol addiction-like behaviors in male or female rats

We then retrospectively assessed whether basal levels of anxiety-like behavior, or social hierarchy (dominant vs. subordinate) could predict alcohol addiction-like behaviors.

**Fig. 3:**
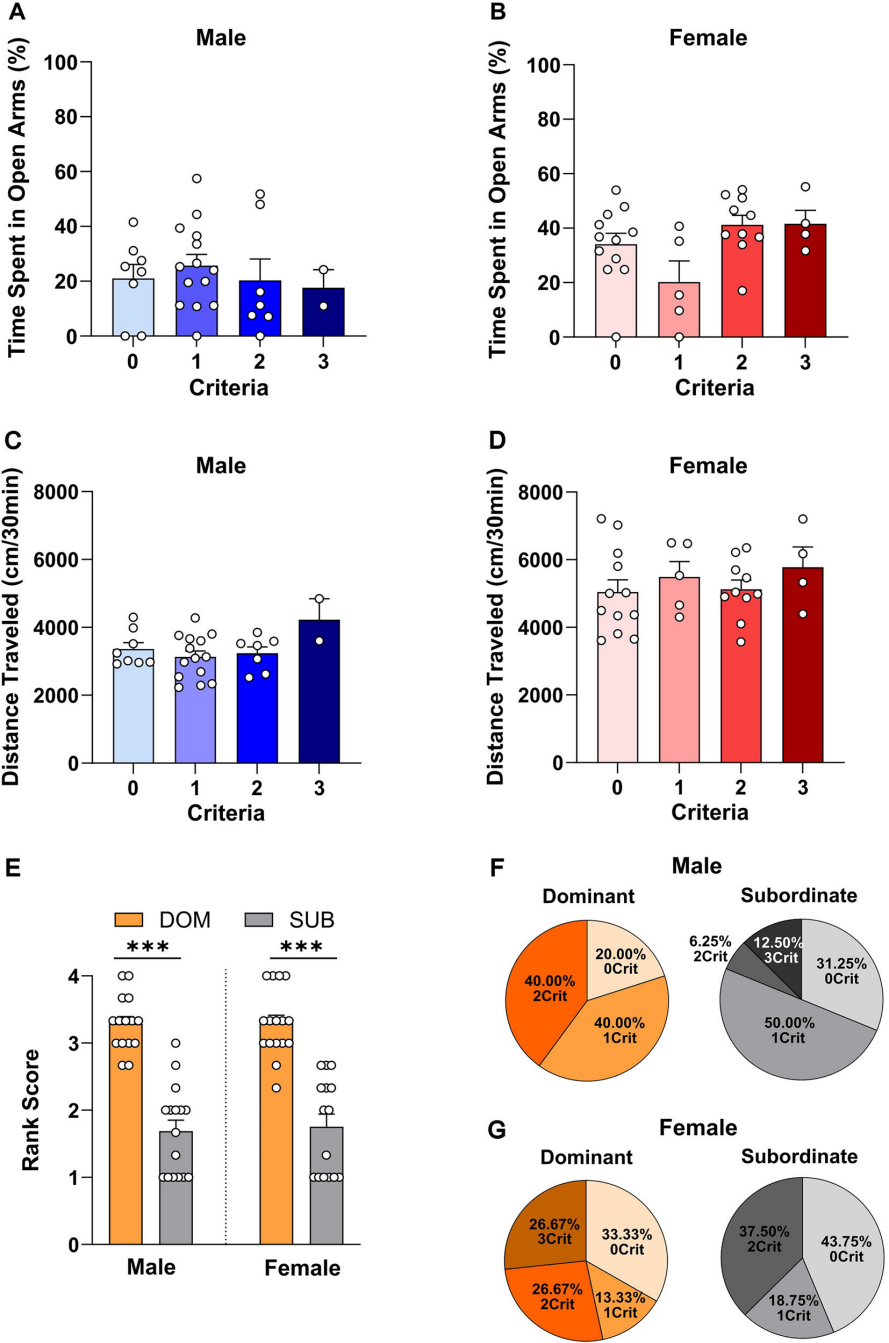
Anxiety and Social Rank Analysis Across Criteria Groups in Male and Female Rats. **A-B** Anxiety-like behavior, measured as the percentage of time spent in the open arms of the Elevated Plus Maze test, in male and female rats (n = 31/sex). **C-D** Locomotor activity, assessed as the distance traveled (cm) in a novel apparatus during the Open Field test, in male and female rats. **E** Rank scores of dominant and subordinate animals, determined based on confrontations won in the Tube test. **F**. Distribution of dominant and subordinate male rats among the four criteria groups. Dominant rats: 0crit: 20.00%; 1crit: 40.00%; 2crit: 40.00%. Subordinate rats: 0crit: 31.25%; 1crit: 50.00%; 2crit: 6.25%; 3crit: 12.50%. **G** Distribution of dominant and subordinate female rats among the four criteria groups. Dominant rats: 0crit: 33.33%; 1crit: 13.33%; 2crit: 26.67%; 3crit: 26.67%. Subordinate rats: 0crit: 43.75%; 1crit: 18.75%; 2crit: 37.50%. Values are presented as mean ± SEM. ***p < 0.001 indicates significant differences between social ranks (dominant vs. subordinate).

Basal anxiety-like state, measured as time spent in the open arm of the elevated plus maze test, did not result altered when comparing groups based on criteria count neither in males (H = 1.318, *p* > 0.05; Fig. 3A) nor in females [F_(3, 27)_ = 3.149, p = 0.041; Fig. 3B]. Furthermore, baseline levels of anxiety-like behavior did not differ between sexes fig. S7A).

Similarly, exploration in the open field test was not associated with criteria count, neither in males (H = 3.125, *p* > 0.05; Fig. 3C) nor in females [F_(3, 27)_ = 0.5745, *p* > 0.05; Fig. 3D]. However, when comparing baseline exploratory behavior between sexes, females explored the environment more than males fig. S7D).

To classify rats as dominant or subordinate, we performed the tube test, which showed that dominant rats consistently scored higher than subordinates in both sexes (males: U = 5, *p* < 0.001; females: U = 6, *p* < 0.001; Fig. 3E and fig. S8A–B). Dominant animals consistently outperformed subordinates in both males fig. S8C) and females fig. S8D). A second tube test conducted at the end of the experiments confirmed the stability of social hierarchy over time (males: r = 0.65, *p* < 0.001; females: r = 0.67, *p* < 0.001; fig. S8E–F). The distribution of animals classified as socially dominant or subordinate in the tube test across the three addiction-like criteria groups is illustrated in Fig. 3F-G.

Lastly, the correlation matrix of various behavioral measures figs. S9 and S10) showed that, in both sexes, neither anxiety-like and exploratory behaviors (assessed in the EPM and OFT) nor social hierarchy were correlated with the animals’ global addiction score.

### Factor analysis identifies sex-specific behavioral dimensions associated with addiction vulnerability

**Fig. 4:**
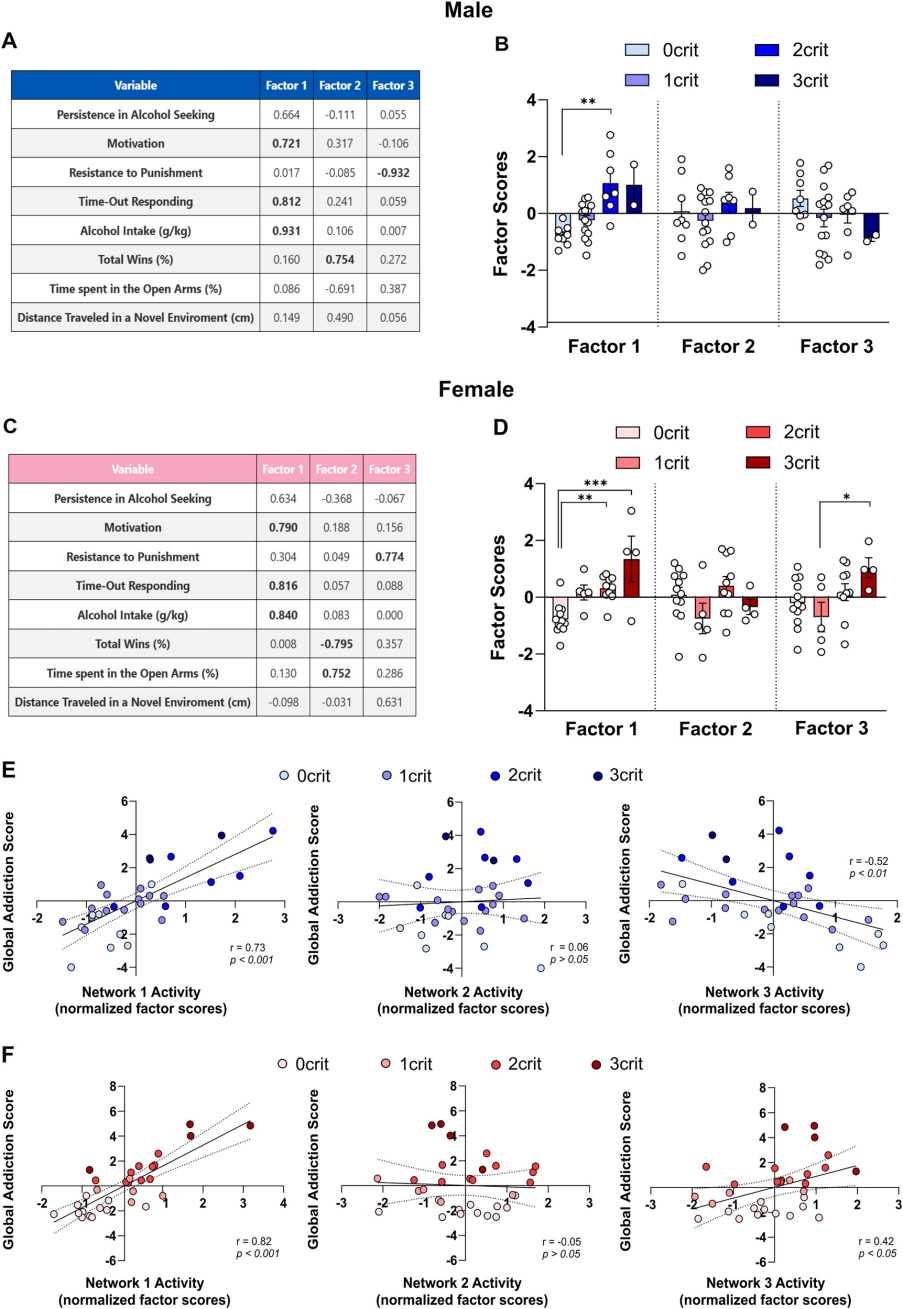
Factor Analysis Reveals Shared and Sex-Specific Behavioral Dimensions underlying Addiction-Like Behaviors. **A-C** Factor loadings according to each factor for male (**A**) and female (**C**) rats (n = 31/sex). **B-D**. Factor scores across animals grouped by the number of addiction-like criteria (0–3crit) in males (**B**) and females (**D**). **E-F** Correlations between global addiction scores and the three extracted factors (labeled Network 1–3) in males (**E**) and females (**F**). Values are presented as mean ± SEM. *p < 0.05, **p < 0.01, ***p < 0.001 indicate significant differences between criteria groups (0crit, 1crit, 2crit, and 3crit).

To better characterize the behavioral dimensions underlying vulnerability to addiction, we performed a principal component extraction followed by factor analysis on a set of behavioral measures related to alcohol-related behaviors, anxiety-like behaviors, and social hierarchy. In both sexes, three factors with eigenvalues greater than 1 were identified. After applying Varimax rotation, the extracted factors accounted for 64.14% of the total variance in males and 64.26% in females. “Factor 1” was the primary dimension in both sexes (35.03% variance in males, 32.88% in females), marked by strong loadings for alcohol intake, motivation, and time-out responding, persistence in alcohol-seeking also contributed but with lower loading. “Factor 2” showed sex differences: in males (15.27% variance), it was defined by a positive loading for tube test wins; in females (17.11%), by a negative loading for tube test wins and a positive loading for time in open arms (EPM test). “Factor 3” also differed by sex: in males (13.84%), it had a strong negative loading for resistance to punishment; in females (14.27%), a positive loading for the same variable, with moderate contribution from distance traveled in a novel environment. Factor loadings are shown in Fig. 4A–C.

To assess whether individual differences in these behavioral dimensions relate to addiction vulnerability, we plotted factor scores by the number of addiction-like criteria met. Rats meeting more criteria showed higher “Factor 1” scores in both males (Fig. 4B) and females (Fig. 4D). Additionally, “Factor 3” scores increased with addiction-like traits in females, while showing a negative trend in males.

We then examined correlations between the three factors and the global addiction score. “Factor 1” positively correlated with GAS in both sexes (males: r = 0.73, *p* < 0.001, Fig.4E; females: r = 0.64, *p* < 0.001, Fig. 4F). Notably, “Factor 3” was negatively associated with GAS in males (r = –0.52, *p* < 0.07, Fig. 4E) and positively in females (r = 0.42, *p* < 0.05, Fig. 4F). No significant correlation emerged between Factor 2 and GAS in both sexes (Fig. 4E-F).

## Discussion

This study investigated sex differences in vulnerability to AUD using a multi-symptom model of alcohol addiction. We found significant sex-specific mechanisms underlying AUD vulnerability, potentially offering insights for understanding sex differences in AUD and developing targeted treatment strategies.

### Sex differences in alcohol-related behaviors

Our findings reveal sex differences underlying distinct mechanisms that influence alcohol self-administration, motivation, and persistence in alcohol-seeking behavior despite negative consequences in rats. Males exhibited higher rates of alcohol-reinforced responses across all fixed-ratio self-administration paradigms. The literature on operant alcohol self-administration remains inconsistent with regard to sex differences. While some studies report no such differences [63], others suggest higher self-administration rates in females [64, 65]. However, when intake levels were normalized for body weight, male and female rats consumed a comparable amount of alcohol, indicating that sex does not affect alcohol intake under our conditions. This is consistent with previous studies from our lab [66] and others [62, 64, 67, 68].

Male rats also were characterized by greater impulsive-like behaviors during alcohol self-administration, as indicated by a higher number of active lever responses during the time-out period compared to females. However, this finding may be confounded by the overall higher response rates observed in males during self-administration.

Additionally, males showed higher motivation to respond for alcohol under progressive ratio schedule, consistent with recent findings [62, 63, 69]. However, other studies have reported higher motivation in females under similar progressive ratio conditions and alcohol concentrations [70], and additional studies have found no sex differences at all [64, 71]. Interestingly, we show here that resistance to punishment is not affected by sex, which contrasts with recent reports suggesting higher compulsivity in females [62, 70]. These inconsistencies across studies may reflect methodological variations or differences in rat strain. For example, Toivanen et al. [62] assessed individual variability in punishment-resistant alcohol self-administration by gradually increasing footshock intensity [62], thereby capturing a more nuanced profile of compulsivity. McDonald et al. (2024), on the other hand, used the same shock intensity as our study but applied it over a longer period [69], potentially enhancing the detection of punishment-resistant behavior. Notably, their protocol also included a four-week home-cage intermittent two-bottle choice procedure prior to the punishment phase, which may have fostered stronger alcohol preference or more stable intake patterns. Such procedural differences likely contribute to the observed discrepancies in the proportion of animals classified as compulsive across studies.

Taken together, and partly overlapping with the findings reported by Toivanen et al. [62], our findings suggest that males are more influenced by motivation and impulsivity, while females are more affected by emotional and stress-related factors, underscoring the need for sex-specific approaches to understanding and treating AUD.

### Different incidence of addiction-like phenotype in male and female rats

Using the multi-symptom model of AUD, we classified subjects into four distinct categories based on their criteria count. We found that after prolonged alcohol self-administration, only a minority of both male and female rats met all three addiction-like criteria assessed (persistent alcohol-seeking despite unavailability, high motivation for alcohol, and continued responding despite punishment). In males, our results align with previous studies, though with a lower percentage of individuals meeting all three criteria (6.45 vs. ~12%) [49–51]. These discrepancies may arise from differences in behavioral protocols. Notably, rats in our study were alcohol- and operant-naïve prior to training, whereas earlier studies employed methods such as intermittent two-bottle choice alcohol consumption in the home cage [49, 51] or saccharin fading [50] to facilitate operant acquisition. Given that prior alcohol exposure enhances vulnerability to developing AUD-like behaviors [70, 71], it is plausible that this factor contributed to the higher proportion of rats meeting 3 criteria reported in previous studies. Supporting this notion, we show here a robust correlation between alcohol consumption history and the global addiction score (GAS) in both sexes, reinforcing the notion that consumption is a major factor driving AUD vulnerability [49–51]. These findings align with clinical evidence suggesting that heavy alcohol consumption is a critical factor in the development of AUD [71, 72], but it is not sufficient on its own and interacts with genetic, neurobiological, and environmental factors that heighten individual vulnerability [73]. Furthermore, differences in alcohol concentrations compared to previous studies [49–51] may partly account for the variability in behavioral findings and hinder direct cross-study comparisons. Another potential explanation for the lower percentage of individuals meeting all three criteria in our study lies in the use of group housing throughout the experimental protocol. While this approach was chosen to maintain a more ethologically valid and less stressful environment, and to enable the assessment of social dominance effects on vulnerability to alcohol addiction-like behaviors, it is important to acknowledge that social housing itself may have influenced behavioral outcomes. Social interactions can serve as a form of environmental enrichment, buffering stress responses and thereby modulating alcohol intake and the expression of addiction-like behaviors [74, 75]. Furthermore, the presence of cage-mates may have introduced variability in individual trajectories through mechanisms such as social facilitation or inhibition. Finally, we acknowledge that assessing anxiety-like behaviors at multiple time points, particularly after prolonged alcohol exposure, could have provided additional insights into the dynamic relationship between anxiety and alcohol-seeking behaviors. Future studies should therefore directly compare group- and single-housed conditions to better disentangle the contribution of social context to addiction vulnerability and progression, and to longitudinally explore how fluctuations in anxiety-like behaviors interact with alcohol consumption and social environment.

Adding another layer of complexity, to the extent of our knowledge, this is the first report employing the multi-symptom model of AUD in female rats. We found that, compared to males, a larger proportion of female rats met all three criteria (12.90%), indicating that females might be more sensitive to develop an AUD-like phenotype. However, this finding contrasts with epidemiological data showing that men are more frequently diagnosed with AUD [76]. One potential explanation for this discrepancy lies in cultural influences. It has been suggested that the historically higher prevalence of AUD in men may partially stem from societal norms and stigma directed at women who drink, which have shaped drinking behaviors across human populations [77]. Future studies should assess the model’s translatability to human conditions by incorporating both sexes and refining the experimental protocol to better capture sex differences in prevalence, and to figure out whether this model better reflects certain phenotypic profiles in one sex over the other.

### Sex differences in behavioral dimensions of alcohol addiction-like phenotype

Emerging evidence suggests that the neural mechanisms underlying the transition to AUD may differ between sexes [78]. Our factor analysis revealed both shared and sex-specific dimensions contributing to addiction vulnerability. “Factor 1”, which captured persistence in alcohol-seeking, motivation, alcohol intake, and impulsivity, emerged as a common component in both sexes and was positively associated with the GAS, supporting its role as a core feature of addiction-like behavior. In contrast, “Factor 3” showed opposite loadings for resistance to punishment across sexes and was differentially correlated with the GAS. In males, it was primarily defined by a strong negative loading on resistance to punishment, potentially reflecting behavioral inhibition or sensitivity to aversive outcomes. In contrast, in females, “Factor 3” was positively loaded by both resistance to punishment and exploratory activity, which may suggest a more disinhibited, novelty-seeking profile. While speculative, this sex divergence in behavioral composition and its opposing associations with the GAS support the idea that “Factor 3” may index distinct addiction-relevant traits in males versus females.

Importantly, resistance to punishment is loaded independently onto a separate factor, distinct from the other alcohol-related measures, indicating that it reflects a distinct dimension within the broader phenomenology of AUD. These findings suggest that this component may contribute to addiction vulnerability in a sex-specific manner, potentially reflecting greater persistence in alcohol use despite adverse consequences in females. Consistent with this data, we observed pronounced sex differences in resistance to punishment and its relationship with AUD vulnerability. In females, we found that 3 criteria animals (AUD-vulnerable) self-administered higher amount of alcohol despite negative consequences, in contrast to their 0 criteria (AUD-resilient) counterparts. In contrast, in males, we observed no significant difference in punishment resistance between 0 criteria and 3 criteria groups. This absence of effect may, at least in part, be attributed to the limited number of males meeting all three addiction-like criteria, which likely reduced statistical power to detect group differences. Importantly, the lack of punishment resistance in this group raises the possibility that their addiction score is primarily driven by heightened motivation and persistence in alcohol-seeking, rather than compulsive intake. This interpretation calls into question the AUD-like status of these individuals and may partly account for the sex differences observed in our factor analysis. These results suggest that the underlying behavioral architecture contributing to addiction-like vulnerability may differ between sexes, and that individual traits such as social dominance or anxiety could be associated with distinct components of the addiction-like phenotype in males versus females. These interpretations should be considered with caution, as the classification framework may not fully capture phenotypic variability in males, and further studies with larger samples are needed to validate these findings. Importantly, it is worth noting that these sex differences cannot be attributed to variations in pain sensitivity, as male and female rats displayed comparable footshock thresholds. Moreover, prior alcohol consumption did not account for these differences, as no correlation was found between alcohol intake and resistance to punishment. This latter result aligns with previous reports [42, 59, 60, 69], further strengthening this evidence.

Despite increasing interest in the neural and behavioral mechanisms of compulsive alcohol use, most rodent studies have been conducted exclusively in males [42, 59, 60, 79–82]. Recent research, however, has begun to address this gap. Studies in Wistar [62] and in Long-Evans [69] rats showed that females engage in higher levels of punished alcohol self-administration compared to males. Similarly, female mice persisted in alcohol use under punishment conditions, tolerating higher shock intensities than males [83, 84], and a greater proportion of females were found in the aversion-resistant group when quinine-adulterated alcohol was used [85, 86]. Our findings align with these recent studies, supporting the notion that addiction-vulnerable female rodents are more resistant to punishment than males, and this phenomenon appears consistent across various aversive conditions. These sex differences in punishment-resistant alcohol use may reflect distinct neurobiological adaptations. Rodent studies conducted exclusively in males have shown enhanced recruitment of brain regions implicated in stress and reward processing, including the central amygdala (CeA), bed nucleus of the stria terminalis (BNST), nucleus accumbens (NAc), periaqueductal gray (PAG), and insular cortex, during compulsive-like alcohol intake induced by quinine adulteration [60, 81, 87]. In the context of footshock punishment, a Fos-mapping study reported that increased activity in the CeA, NAc, and PAG was strongly associated with punishment-resistant alcohol self-administration [42]. More recently, evidence has begun to highlight female-specific mechanisms. Notably, one study [69] showed that a larger proportion of female rats develop compulsive-like alcohol intake following footshock punishment, and that this phenotype is linked to hypofunction of the NAc shell. Together, these findings suggest that distinct neurobiological processes may drive compulsivity in females and underscore the need for further studies to clarify the sex-specific circuit- and molecular-level mechanisms underlying the development and persistence of compulsive alcohol use.

### Anxiety-like behaviors and social hierarchy do not predict vulnerability to developing AUD

A retrospective analysis of traits associated with AUD vulnerability showed that anxiety, locomotor activity in a novel environment, and social hierarchy are not correlated with the addiction scores of the animals, suggesting that these factors do not play a role in the vulnerability to develop alcohol addiction-like behaviors in either sex. Accordingly, we previously found that locomotor reactivity to novelty does not predict alcohol choice over a sweet alternative reward or compulsive-like alcohol drinking [43]. The literature on the predictive value of anxiety-related traits for future development of AUD-like behaviors is mixed. Some studies report a strong correlation between anxiety-like behaviors and alcohol intake [88, 89], while others have found either a negative [90, 91] or a null [92] correlation between these factors [90–92]. These discrepancies may stem from differences in experimental protocols, strain-specific factors, or the complex bidirectional relationship between anxiety and alcohol use, where alcohol can both alleviate and exacerbate anxiety symptoms depending on the context and individual vulnerability.

In contrast, the potential influence of social dominance on alcohol-related behaviors in rodents remains largely unexplored. A previous study reported that subordinate male rats consumed more alcohol than their dominant counterparts when given free access to alcohol [93]. However, methodological differences in assessing both social hierarchy and alcohol-related behaviors prevent direct comparisons with our findings. Most insights on this topic come from studies in non-human primates, with mixed results: while one study found that social subordination was associated with increased alcohol consumption [94], another failed to replicate this effect [95]. In our study, correlation analyses did not show significant associations between social hierarchy and either the three addiction-like criteria or other alcohol-related behaviors. These findings suggest that social dominance, at least in rats, may not play a major role in AUD vulnerability. However, further studies are needed to comprehensively evaluate the influence of social hierarchy on alcohol-related behaviors and its potential contribution to the development of AUD.

Since impulsivity has been identified as a predictor of punishment-resistant drug-taking [96], we examined whether impulsive traits correlate with AUD-like behaviors. Our data shows that while 0 criteria and 1 criteria groups decreased their responses during time-out periods over sessions during self-administration training, 3 criteria and 2 criteria rats maintained a sustained rate of lever pressing during timeout periods throughout acquisition. These data suggest that higher impulsivity might be predictive of future development of AUD-like traits. This conclusion is further supported by our correlation matrix, which showed that in both sexes, time-out responding is highly correlated with the GAS. However, the interpretation of this behavioral readout is complex. In our protocol, active lever presses during the 10 s time-out period were operationally defined as “impulsive-like behavior” [51]. Yet, in this context, time-out responding may reflect multiple overlapping dimensions. On one hand, it may index persistent alcohol-seeking, a hallmark of addiction-like behavior often linked to habit formation and reduced behavioral flexibility [97, 98]. On the other hand, it may also reflect impulsivity or altered reward expectancy, particularly in animals with heightened motivation for alcohol [99]. Therefore, rather than representing a pure measure of trait impulsivity, time-out responding might better be understood as reflecting a broader spectrum of maladaptive behavioral strategies that are characteristic of individuals with greater vulnerability to AUD-like behavior. Further studies employing dedicated tasks specifically designed to assess impulsivity are needed to disentangle these dimensions and to more accurately characterize impulsive traits in AUD-resilient versus AUD-vulnerable rats.

Although our study did not reveal any single behavioral dimension that robustly predicted vulnerability in either males or females, our findings highlight behavioral sex differences in the addiction-like phenotype. In this study, resistance to punishment played a key role in shaping the vulnerability to AUD in females suggesting that emotional and stress-related traits may be relevant in driving addiction-like behaviors in this sex. In contrast, the elevated active lever responding during time-out periods and the higher motivation for alcohol observed in males may reflect impulsivity- and reward-driven features that contribute to addiction vulnerability in males. These findings partly replicated the work of Toivainen et al. [62] that identified corticosterone levels and pain sensitivity as predictors of compulsive alcohol self-administration in females, whilst motivation for seeking alcohol as the main predictor of this phenotype in males. The behavioral sex differences observed in our study align with patterns reported in clinical populations. For instance, women affected by AUD tend to display higher rates of comorbid psychiatric disorders, such as anxiety and depression, compared to men [100]. They are also more likely to use alcohol as a coping mechanism for managing negative emotions, showing increased relapse risk [30, 31]. Conversely, men more frequently report drinking in social contexts or in pursuit of positive emotional experiences [27, 28]. These cross-species parallels not only reinforce the translational relevance of our findings but also underscore the need for future research to more precisely define these sex-specific predictive traits and uncover their underlying neurobiological mechanisms.

## Conclusion

This study underscores the importance of considering sex-specific mechanisms when studying AUD. To our knowledge, we are the first to adapt a multi-symptom model to investigate the contributing factors to AUD in both male and female rats. The greater vulnerability observed in females, driven by unique behavioral traits, such as resistance to punishment, highlights the possible need for tailored approaches in addiction research. These results emphasize the complexity of factors contributing to addiction vulnerability and call for further exploration of sex differences in preclinical models to better understand the underlying mechanisms of AUD.

## Supplementary information

Supplementary Materials

## Data Availability

The data used to generate results of this study are available from the corresponding authors upon request.
