# Supplementary material for: Sex-Specific patterns of vulnerability to alcohol addiction-like behaviors in rats
Source: Transl Psychiatry. 2026 Jan 29;16:59. doi: 10.1038/s41398-026-03825-w (PMC12873331; doi:10.1038/s41398-026-03825-w)
Supplement: Supplementary file 1 — Supplementary Materials [file 41398_2026_3825_MOESM1_ESM.docx]

**SUPPLEMENTARY MATERIALS**

**Sex-Specific Patterns of Vulnerability to Alcohol Addiction-like Behaviors in Rats**

**Running title:** Sex Differences in Alcohol Addiction Vulnerability

Anna Maria Borruto^1†*^, Andrea Coppola^1†^, Leon Höglund^1^, Sandra Eriksson Solander^1^, Michele Petrella^1^, Markus Heilig^1^, and Eric Augier^1*^

*^1^Center for Social and Affective Neuroscience, Department of Biomedical and Clinical Sciences, Linköping University, S-581 85, Linköping, Sweden*

*^†^These authors contributed equally to this work.*

***Corresponding authors**:

Dr. Anna Maria Borruto, Center for Social and Affective Neuroscience, Department of Biomedical and Clinical Sciences, Linköping University, S-581 85, Linköping, Sweden.

E-Mail: [anna.maria.borruto@liu.se](mailto:anna.maria.borruto@liu.se)

Dr. Eric Augier, Center for Social and Affective Neuroscience, Department of Biomedical and Clinical Sciences, Linköping University, S-581 85, Linköping, Sweden.

Telephone: [+4613282995](tel:+4613282995)

E-Mail: [eric.augier@liu.se](mailto:eric.augier@liu.se)

**MATERIALS AND METHODS**

**Animals**

Male (n = 32) and female (n = 32) Wistar rats (Charles River, Germany) were used in this study. At the start of the experiment, 8-week-old rats weighed 270-300 g (males) and 190-220 g (females). They were housed in groups of four in individually ventilated cages (NextGen 1800, Allentown Europe Ltd, UK) with conventional wood chips bedding from the animal facility at Linköping University. The rats were maintained in a pathogen free-environment on 12-hour light/dark cycle (lights off at 7:00 AM and on at 7:00 PM), with controlled temperature (20-22°C) and humidity (45-55%) regulated by an automated system (Allentown Ecoflo, Allentown Europe Ltd, UK). Food (diet A04, SAFE, Rosenberg, Germany) and water were available ad libitum. All experiments were conducted during the dark phase of the light/dark cycle. Upon arrival, the animals were left undisturbed to habituate to the new housing conditions for one week, after which, they were handled for five days to familiarize with the experimenters before any procedures were started. Animal care and experimental procedures were carried out in accordance with the European Union Directive 2010/63/EU and Swedish laws. The protocol was approved by the Swedish Animal Ethics Committee (Jordbruksverket, Dnr 01680-2020 ID1942). Every effort was made to minimize the number of animals used and their discomfort, in adherence to the 3R principles for animal welfare.

**Elevated Plus Maze test**

To assess basal anxiety-like behavior, animals underwent elevated plus maze test (1), where spontaneous exploration of open versus enclosed spaces served as the primary measure. The apparatus consisted of two open arms (50 cm x 10 cm) and two enclosed arms (50 cm x 10 cm x 40 cm), arranged in opposing pairs, with a central neutral zone (10 cm x 10 cm). The maze was elevated 70 cm above the floor and placed in a sound-attenuated room illuminated with red light (approximately 30 lux). A camera (Basler aca1300, Noldus Technology, The Netherlands) was positioned above the maze and was used to acquire recordings for behavioral scoring. The time spent in the open arms of the maze was automatically recorded using a video-tracking software (Any-Maze, Stoelting, USA). A rat was considered within the zone of the apparatus when at least two of its paws were inside it.

**Open Field test**

To evaluate basal exploratory behavior and locomotory reactivity to novel environments, the animals underwent an open field test. They were placed in the center of an open field chamber (43.2 x 43.2 x 30.5 cm) (ENV-515S-A, MedAssociates, Vermont, USA) and allowed to explore the arena for 30 minutes. The chamber was located in a sound-attenuated cubicle with red light illumination (approximately 30 lux). It was equipped with 16-beam infrared (IR) arrays on both the X and Y axis to enable automated tracking of the animal’s position. The total distance travelled in the arena was automatically calculated using the dedicated software (Activity Monitor, MedAssociates, Vermont, USA).

**Tube test**

To determine the animals’ social status, we used the confrontation tube test (2, 3), in which the performance of the animals in pushing a cage mate out of a plexiglass tube serves as a proxy of social dominance. The tube was custom-made (female: 1 m in length and 5 cm inner diameter; male: 1 m in length and 6 cm inner diameter) and was designed to allow only one rat to pass through at a time. To minimize the effects of stress and novelty-induced anxiety, all animals underwent an initial habituation phase to the apparatus, during which they were allowed to freely cross the tube for 10 minutes per day. The habituation was considered complete when the latency to the first crossing became negligible. After habituation, the animals entered the test phase, which consisted of a series of confrontations between cage mates from the same group in a round-robin design. In the start of each confrontation, two rats were allowed to enter the tube from opposing ends. Once they met in the middle, a gate was lifted to allow interaction between the animals. Eventually, one of the animals would push the other out of the tube and would be deemed as the “winner” of the confrontation. Each confrontation was repeated three consecutive times, with the starting side switched every time, to avoid side bias. The series of confrontations was repeated for three consecutive days to assess the stability of the winning phenotype. The total number of won confrontations was used to assign a rank score, and animals were ranked from the highest to lowest score (α > β > γ > ω). The two animals with the highest rank scores were classified as dominant, while those with the lowest rank scores were classified as subordinate. The tube test was repeated in two points of the experimental design: at the beginning of the experiment, prior to any alcohol self-administration training, and at the end of the experiment, following classification based on the addiction-like criteria. Rank scores in the two trials were compared to confirm the stability of the dominance-like phenotype.

**Drugs**

The alcohol solution was prepared by mixing absolute ethanol (Solveco, Rosersberg, Sweden) with tap water to achieve a final concentration of 20% (v/v) alcohol.

**Operant alcohol self-administration**

Operant- and drug-naïve rats were trained to self-administer 20% (v/v) alcohol without prior sucrose or saccharin fading, as previously described (4, 5). Alcohol self-administration sessions were conducted in modular rat chambers (Med Associates, VT, USA) housed within sound-attenuating, ventilated cubicles. Each chamber was equipped with two retractable levers on the front panel, with cue lights positioned above each lever, and a house light located at the top of the chamber, opposite the levers. At the start of each self-administration session, the levers were extended. Alcohol was delivered via syringe pumps (Med Associates, VT, USA) connected to polyethylene tubing that led to a receptacle. Chamber settings and reinforcement schedules were managed by MED-PC IV software (Med Associates, VT, USA), which also recorded the number of operant responses on both levers and the total number of reinforcers obtained.

**Evaluation of the Three Criteria for AUD Like-Behavior**

Rats underwent a total of 60 alcohol self-administration training sessions, conducted once daily, 5–6 days per week. Each 30-minute session comprised a 10-minute reward-available period (drug period), followed by a 10-minute reward-unavailable period (no-drug period), and concluded with a second 10-minute drug period. During drug periods, completion of the active lever response requirement resulted in the delivery of 0.1 ml of alcohol, accompanied by illumination of the associated cue light for 5 seconds. Following each reward delivery, a 10-second timeout period was imposed, during which additional responses to the active lever had no programmed consequences. However, responses to both levers during the timeout were recorded as a measure of impulsive-like behavior (6). During the no-drug period, signalled by the illumination of the house light, responses to the active lever were not rewarded. Inactive lever responses were recorded throughout the entire session but had no programmed consequences. Training began on a fixed-ratio 1 (FR1) schedule of reinforcement, requiring one active lever response per reward. Once stable responding was achieved (session 23), animals progressed to an FR2 schedule (sessions 24–36) and then to an FR3 schedule (sessions 37–60). Two rats (1 female and 1 male) were excluded from the study due to failure to acquire alcohol self-administration behavior (≤3 active lever presses).

**Persistence in alcohol-seeking**

To measure persistence in alcohol-seeking behavior, we recorded the number of responses on the active lever during the no-drug periods across all training sessions. For the evaluation of addiction-like criteria, the average number of responses from the FR3 training sessions (sessions 37-60) was used to minimize behavioral fluctuations (7).

**Motivation**

As a measure of the motivation to seek for the alcohol reward, we tested the rats under a progressive ratio (PR) schedule of reinforcement (8), where the requirement of active lever responses to obtain the reward was increasing progressively. The sequence of the ratio schedules was as follows: 1, 2, 3, 4, 6, 8, 10, 12, 16, 20, 24, 28, 32, 36 etc. When the ratio requirement was completed, the 0.1 ml alcohol reward was delivered, together with the illumination of the cue light for 5 seconds and a concomitant 5-seconds time-out. The highest ratio completed by the animal during the PR session, referred to as the breakpoint, was used for the final evaluation of addiction-like criteria.

**Resistance to punishment**

To assess continued alcohol use despite negative consequences, rats underwent punished self-administration sessions involving footshock. Initially, animals were exposed to a 10-minute chained schedule, as previously described for the multi-symptom model (7, 9). In this paradigm, the first active lever press under an FR3 schedule triggered the illumination of a distinct green cue light above the lever, signaling the start of the footshock phase. During this phase, a second active lever press delivered a 0.25 mA footshock (0.5 seconds). A third active lever press then resulted in the delivery of the 20% alcohol reward, accompanied by the corresponding cue light and timeout period, as described for unpunished sessions, with the green cue light turning off. If animals failed to complete the sequence within 1 minute, all cue lights were extinguished, and the chain of events restarted.

Since a single 10-minute session may not adequately reveal interindividual differences in punishment-resistant responding (10, 11), a second approach involving multiple sessions was employed. In this paradigm, not every reward cycle was associated with punishment. Adapting a previously established protocol (12, 13), animals received a 0.25 mA footshock (0.5 seconds) every 8th active lever press during the 10-minute drug periods. This ensured that the punishment was not directly contingent upon alcohol reward delivery. Active lever presses during the no-drug periods had no programmed consequences.

In both approaches, footshocks were delivered via the electrified grids of the operant chamber using a shock generator (ENV-414, MedAssociates, VT, USA), controlled by a MED-PC IV script. Rats underwent five punished sessions. We calculated a resistance score to assess the effect of punishment on alcohol self-administration (14-16). This score was defined as: (punished alcohol deliveries)/(punished alcohol deliveries + average of unpunished active lever presses from the last 3 training sessions). The average resistance score from these five punished sessions was used to evaluate how the animals classified within the multi-symptom model.

**Evaluation of addict-like and non-addict-like rats**

A rat was considered positive for a specific addiction-like criterion if its individual score for each addiction-like behavior exceeded the 66th percentile of the total distribution (9, 17). The analysis was conducted separately for males and females, treating the sexes as independent populations. Animals were categorized based on whether they were positive for 0, 1, 2, or 3 criteria. Finally, a Global Addiction Score (GAS) was calculated for each subject as the sum of the normalized z-scores for all criteria (18). Off note, we performed a principal component analysis (PCA) including all addiction-like behavioral measures in male and female rats, considering sex as a factor (Fig. S1). Notably, animals with a higher number of positive addiction-like criteria were consistently located toward the same regions of the PCA space, confirming that the 66th percentile–based classification aligns with the overall behavioral structure revealed by this unbiased multivariate analysis.

**Footshock sensitivity test**

To rule out the possibility that interindividual differences in resistance to punishment were due to variations in the perception of foot shock intensity, animals underwent a footshock sensitivity test, as previously described (19-21). Rats were placed in a modular chamber used for alcohol self-administration training (MedAssociates, VT, USA). Each animal underwent a series of trials, in which they were exposed to a single 0.5-second foot shock, with a 1-minute intertrial interval. The footshock intensity was increased by 0.05 mA in each successive trial. The paw-withdrawal response to the shock was recorded and scored by two blinded observers. The minimum footshock intensity that elicited a 4-paw withdrawal response, resulting in a jump, was recorded as the final readout. The footshock sensitivity test was performed at the end of the experimental timeline, just prior to sacrifice.

**Data analysis**

Statistical analyses were performed using Prism 10 (GraphPad Software Inc., USA), Statistica 13 (TIBCO Software Inc., USA), and SPSS Statistics software (version 29.0.2, IBM Software Inc., USA). Before conducting analyses of variance (ANOVA) or parametric tests, we ensured that the assumptions of homogeneity of variance and normality were met. Homogeneity of variance was assessed with Levene’s test, while the Shapiro-Wilk test was used to evaluate the normality of data distribution. Sex differences in discrimination between active and inactive levers were analyzed using a repeated measures three-way ANOVA, with 'time' as the within-subject factor, and 'sex' and 'lever' as between-subject factors. Similarly, repeated measures two-way ANOVA, with 'time' as the within-subject factor and 'sex' as the between-subject factor, was conducted to assess sex differences in the number of rewards, alcohol intake, and resistance to punishment. The distribution of male and female rats across the four criteria groups and subsequent analyses were conducted as independent populations. Discrimination between active and inactive levers among the criteria groups was examined using a generalized linear mixed model (GLMM). Significant main effects and interactions were further analyzed with pairwise comparisons of means using the Tukey post-hoc test. Group effects in persistence of response, motivation, resistance to punishment, alcohol intake, and global addiction score were analyzed using one-way ANOVA. When the assumption of normality was violated, the non-parametric Kruskal-Wallis test was employed, followed by the Dunn post-hoc test when appropriate. For datasets with comparisons between only two groups, an unpaired t-test was used. If normality was violated, the non-parametric Mann-Whitney test was applied. Factor analysis was performed using principal component extraction followed by Varimax rotation, retaining factors with eigenvalues greater than 1. Principal Component Analysis (PCA) to evaluate the multivariate structure of addiction-like behavioral measures and potential sex-related differences was conducted in Python using the scikit-learn library (v1.5.2). Prior to PCA, data were normalized using the sklearn.preprocessing module, and dimensionality reduction was carried out with sklearn.decomposition.PCA. Additional libraries employed for data handling and visualization included Pandas, NumPy, Matplotlib, and Seaborn (22, 23). The first two principal components (PC1 = 30.28% of variance; PC2 = 18.73% of variance) were subsequently used as dependent variables in multiple linear regression analyses, with sex, number of addiction-like criteria, and their interaction (sex × criteria) as predictors. All correlations were assessed using Pearson’s correlation analysis. All data are expressed as mean ± SEM. The statistical significance was set at *p* < 0.05.


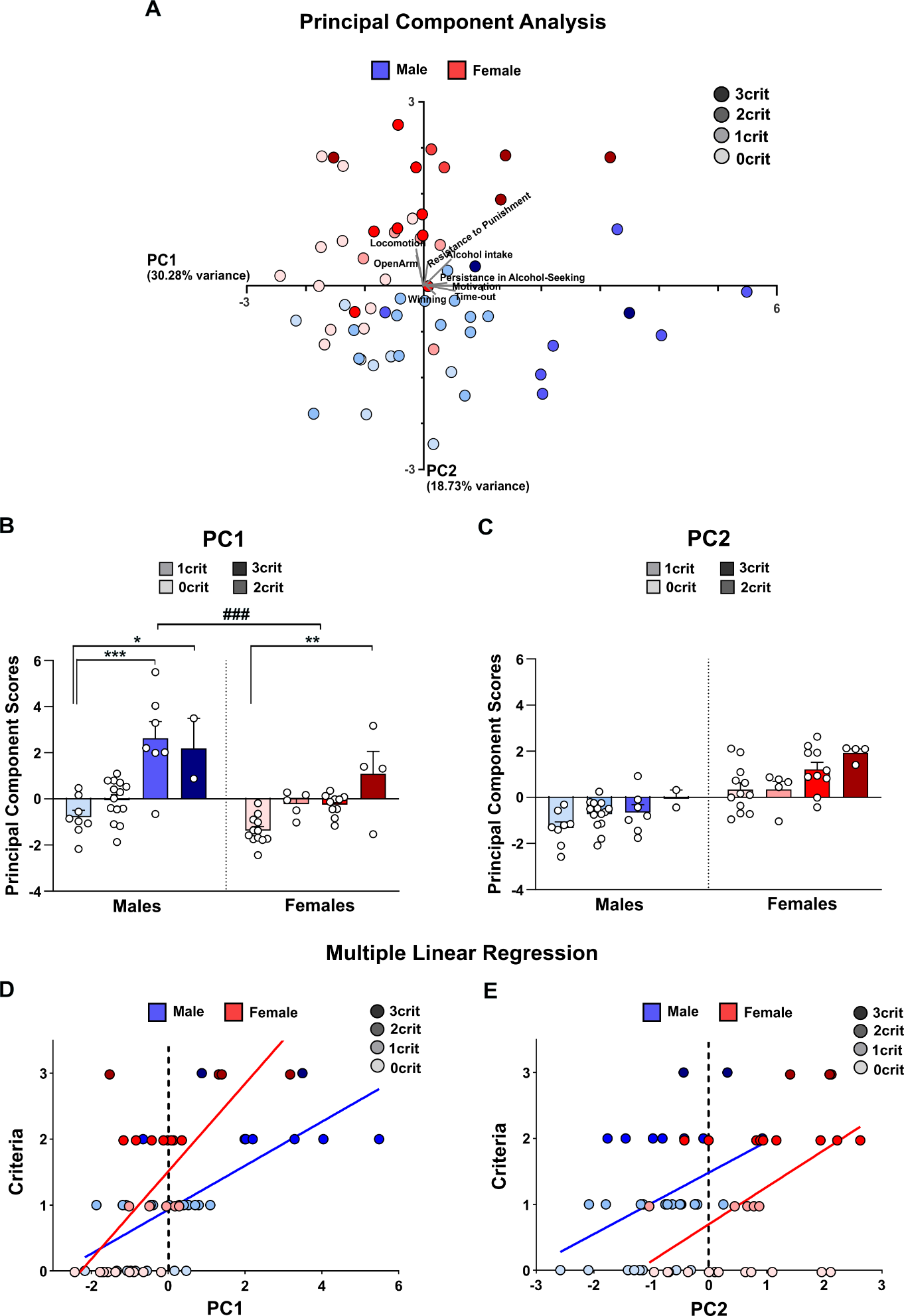


**Figure S1. Principal component analysis (PCA) and multiple linear regression of addiction-like behaviors in male and female rats.**

(**A**) PCA plot showing the distribution of individual animals by sex (blue: males; red: females) and by the number of positive addiction-like criteria (0–3, indicated by the color shading of the circles). The first two principal components (PC1 and PC2) account for 30.28% and 18.73% of the total variance, respectively. Behavioral variables contributing to each component are represented as vectors based on their respective loadings for PC1 and PC2. (**B–C**) Individual and mean (± SEM) PC scores for PC1 (**B**) and PC2 (**C**) across groups defined by the number of addiction-like criteria (0–3crit). (**D–E**) Multiple linear regression analyses using PC1 (**D**) and PC2 (**E**) scores as dependent variables, with sex, criteria, and their interaction (sex × criteria) as predictors. The regression lines illustrate the relationship between criteria and each component within sex. Statistical significance: **p* < 0.05, ***p* < 0.01, ***p* < 0.001 (differences among criteria within each sex); ###*p* < 0.001 (differences between sexes). Statistical analyses are reported in **Table S2**.


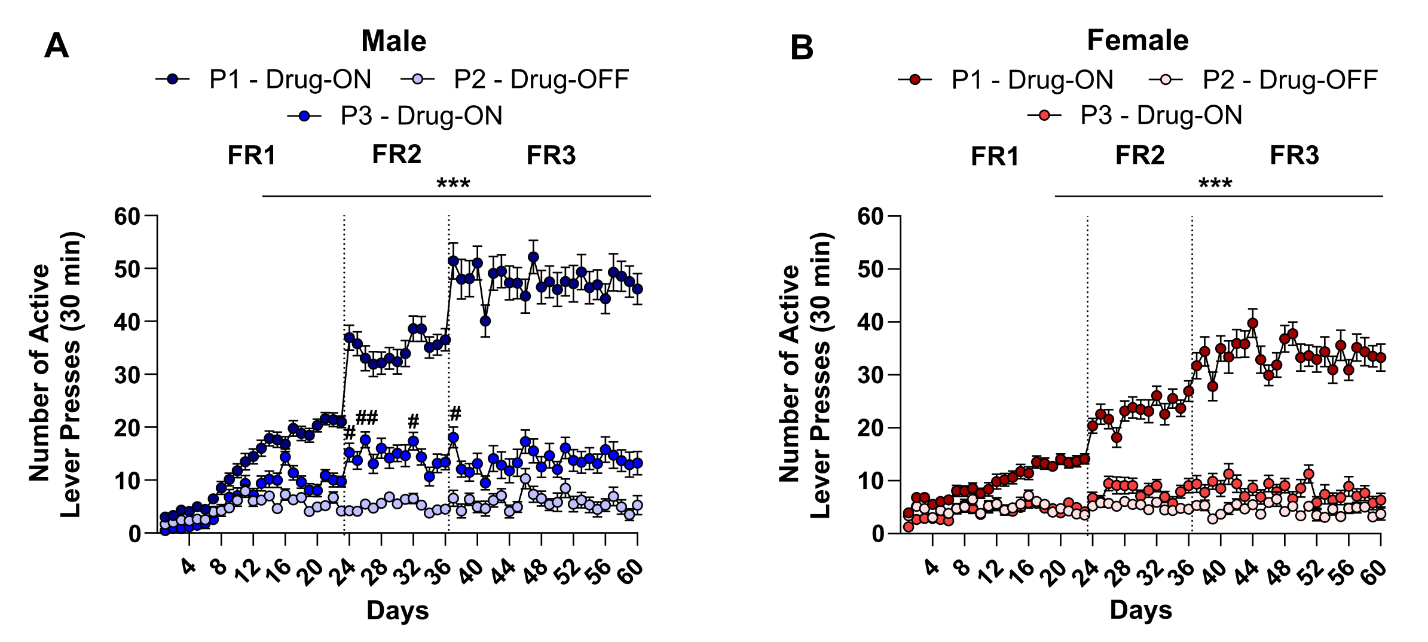


**Figure S2.** Male (**A**) and female (**B**) rats (n = 31/sex) acquired and maintained stable 20% alcohol self-administration levels under a fixed ratio-1 (FR1; sessions 1-23), FR2 (sessions 24-36), and FR3 (sessions 37-60) schedule of reinforcement during daily 30-minute sessions, which were divided into three 10-minute periods: P1 – alcohol available; P2 – alcohol not available; P3 – alcohol available. Values are presented as mean ± SEM. ****p* < 0.01 indicates differences between P1 and P3; #*p* < 0.05, ##*p* < 0.01 indicate differences between P2 and P3. Statistical analyses are reported in **Table S3**.

**
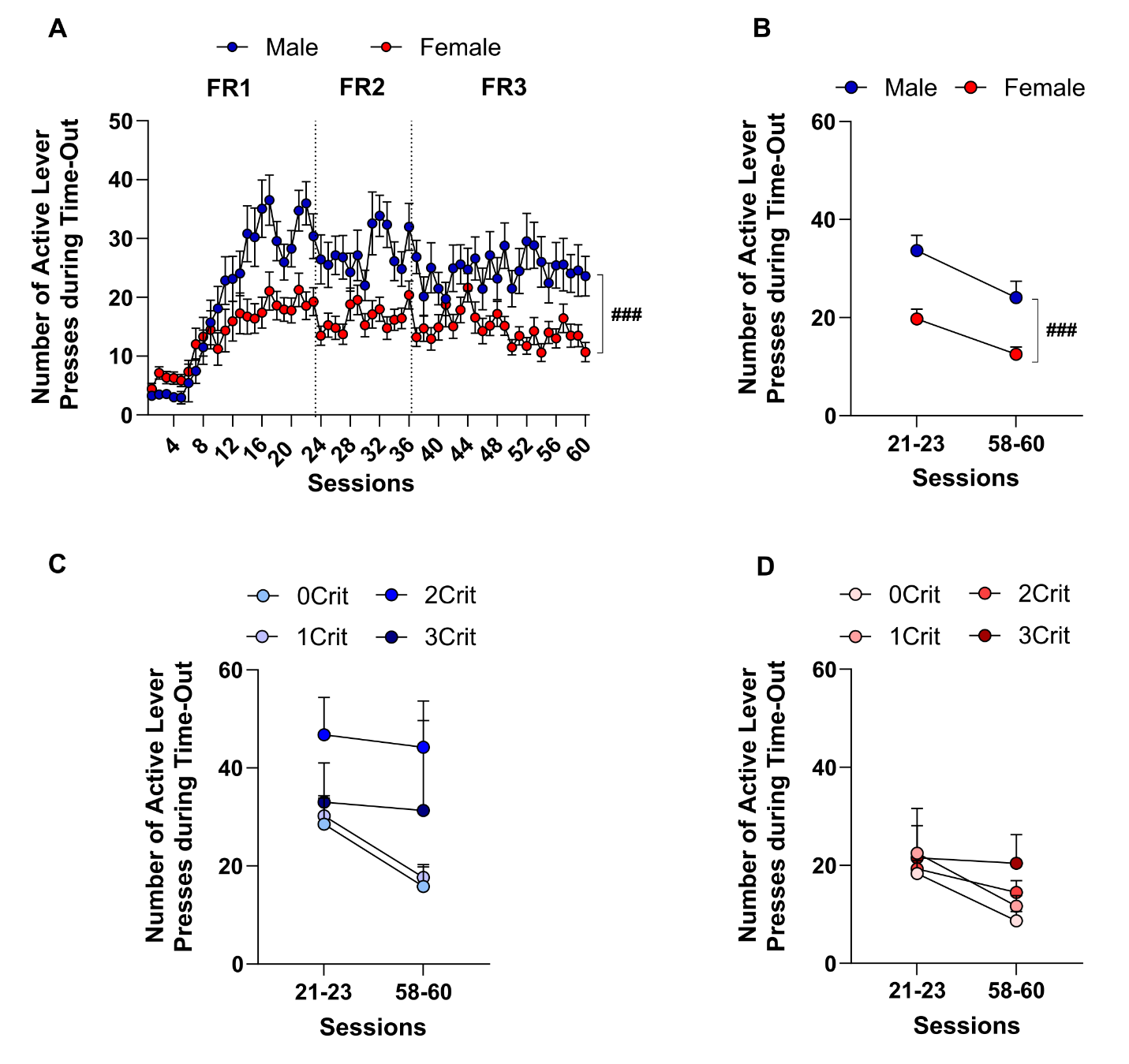
**

**Figure S3.** Number of active lever presses during the 10-seconds time-out in prolonged 20% alcohol self-administration in male and female rats (n= 31/sex) (**A**). Number of active lever presses during the 10-seconds time-out in the initial phase of training (sessions: 21-23) and late phase (sessions: 58-60) in male and female rats (**B**), and among the criteria groups in both male (**C**) and female (**D**) rats. Values are presented as mean ± SEM. ##*p* < 0.01 indicates a difference between sexes (male vs. female). Statistical analyses are reported in **Table S4.**

**
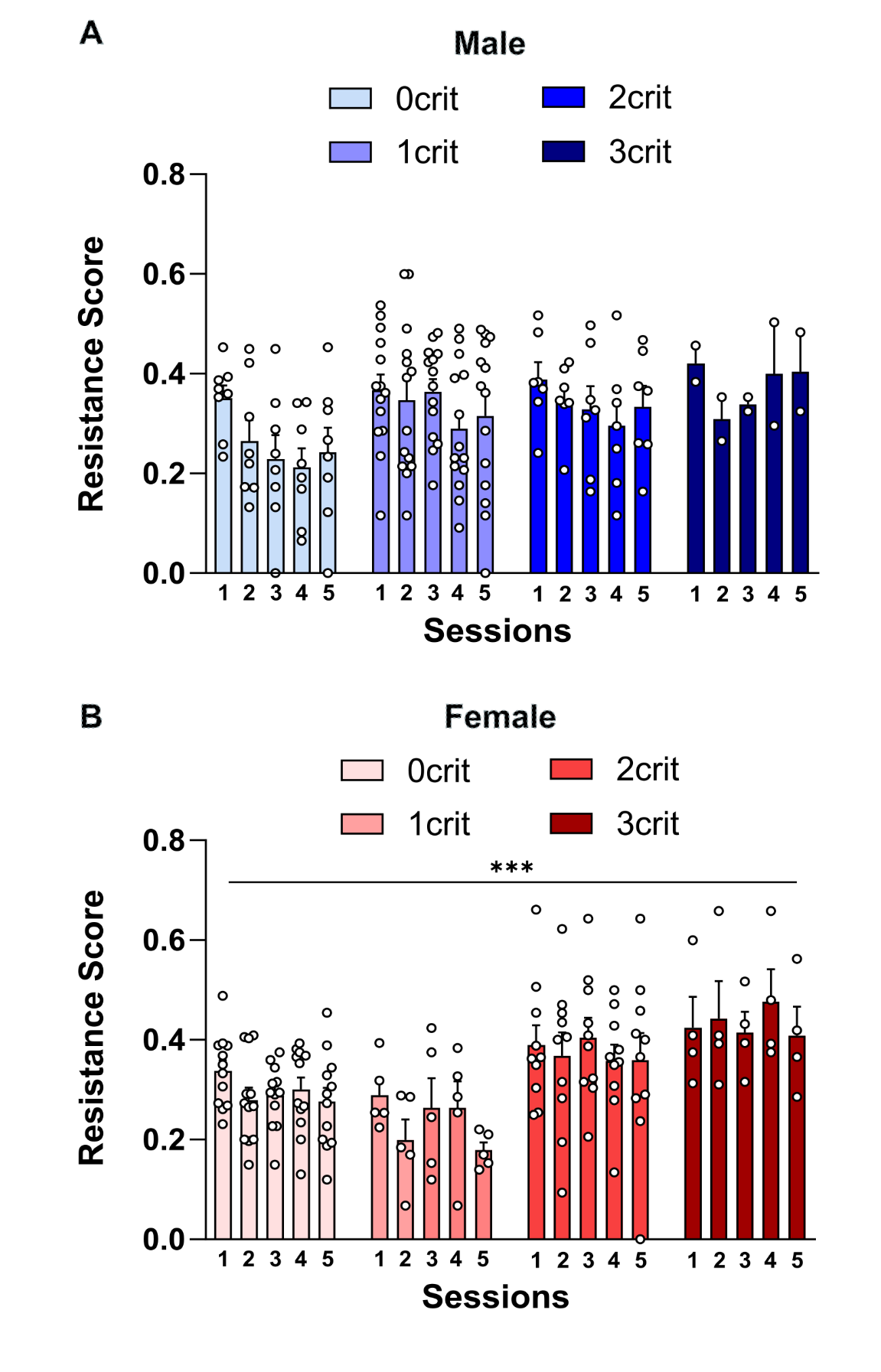
**

**Figure S4.** Resistance score calculated in the five consecutive resistance to punishment sessions and divided by criteria groups in male and female rats (n = 31/sex). Data are presented as mean ± SEM. ****p* < 0.001 indicates the main effect of criteria in female rats. Statistical analyses are reported in **Table S5**.

**
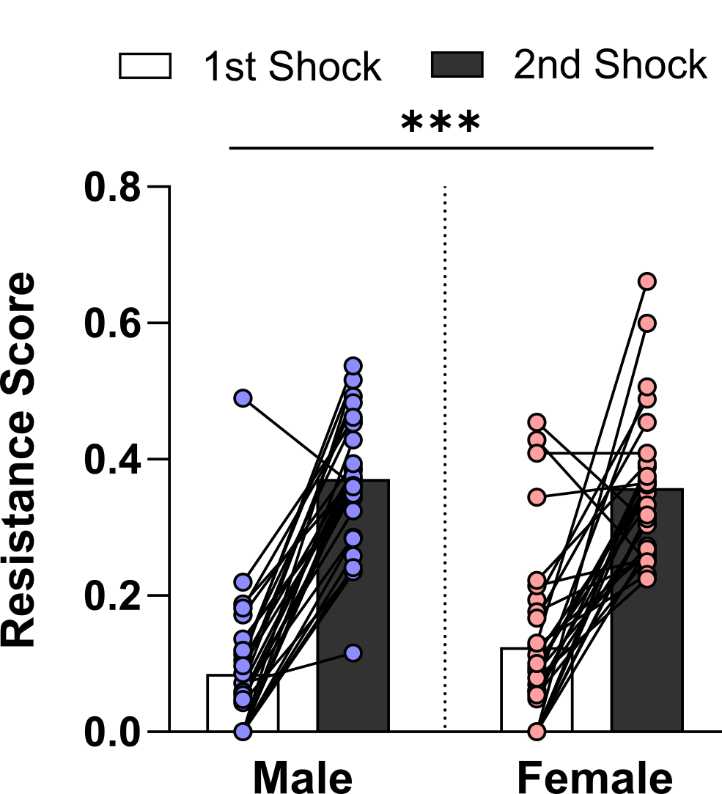
**

**Figure S5.** Resistance scores were measured in male (blue) and female (pink) rats (n = 31/sex) and compared between responses to the first shock protocol (white bars) and the first session of the second shock protocol (black bars). Individual data points are shown, with lines connecting repeated measures from the same subject. Data are presented as mean ± SEM. ****p* < 0.001 indicates a main effect of shock exposure. Statistical analyses are reported in **Table S6**.

**
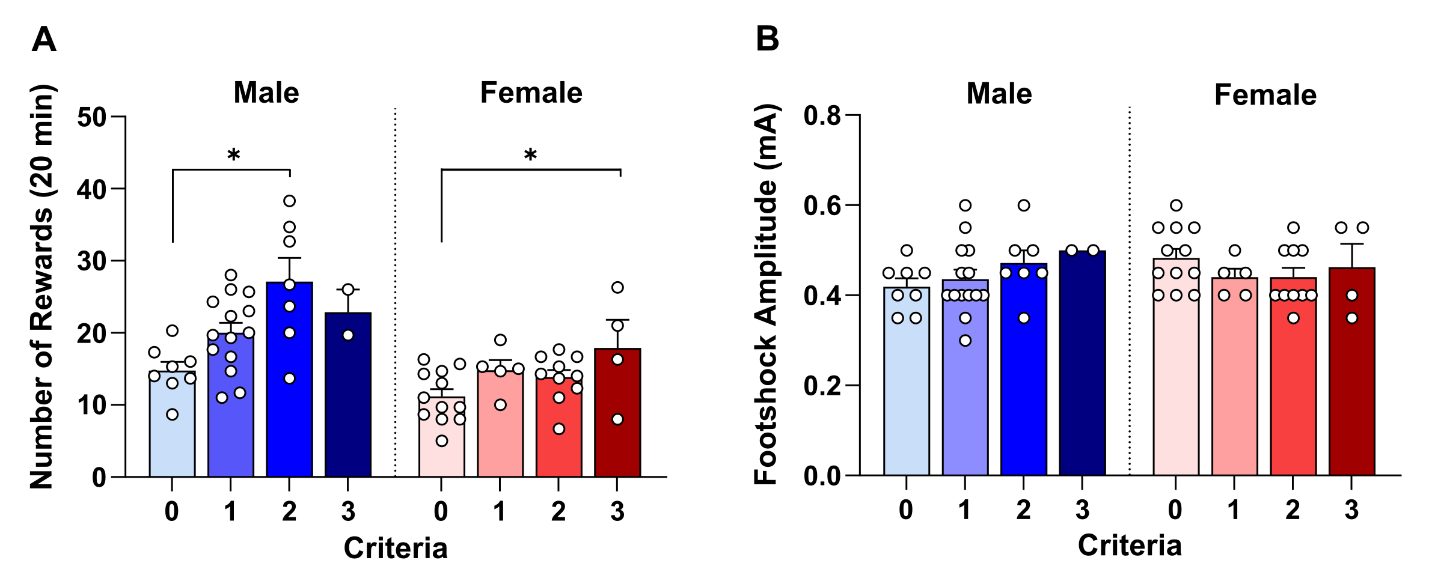
**

**Figure S6.** Number of rewards in the last 3 days (sessions: 58-60) of prolonged 20% alcohol self-administration in male and female rats (n = 31/sex) (**A**). Foot-shock sensitivity test in male and female rats among the different criteria groups (**B**). Data are presented as mean ± SEM. **p* < 0.05 indicates a difference between criteria groups (male: 0 vs. 2; female: 0 vs. 3). Statistical analyses are reported in **Table S7.**

**
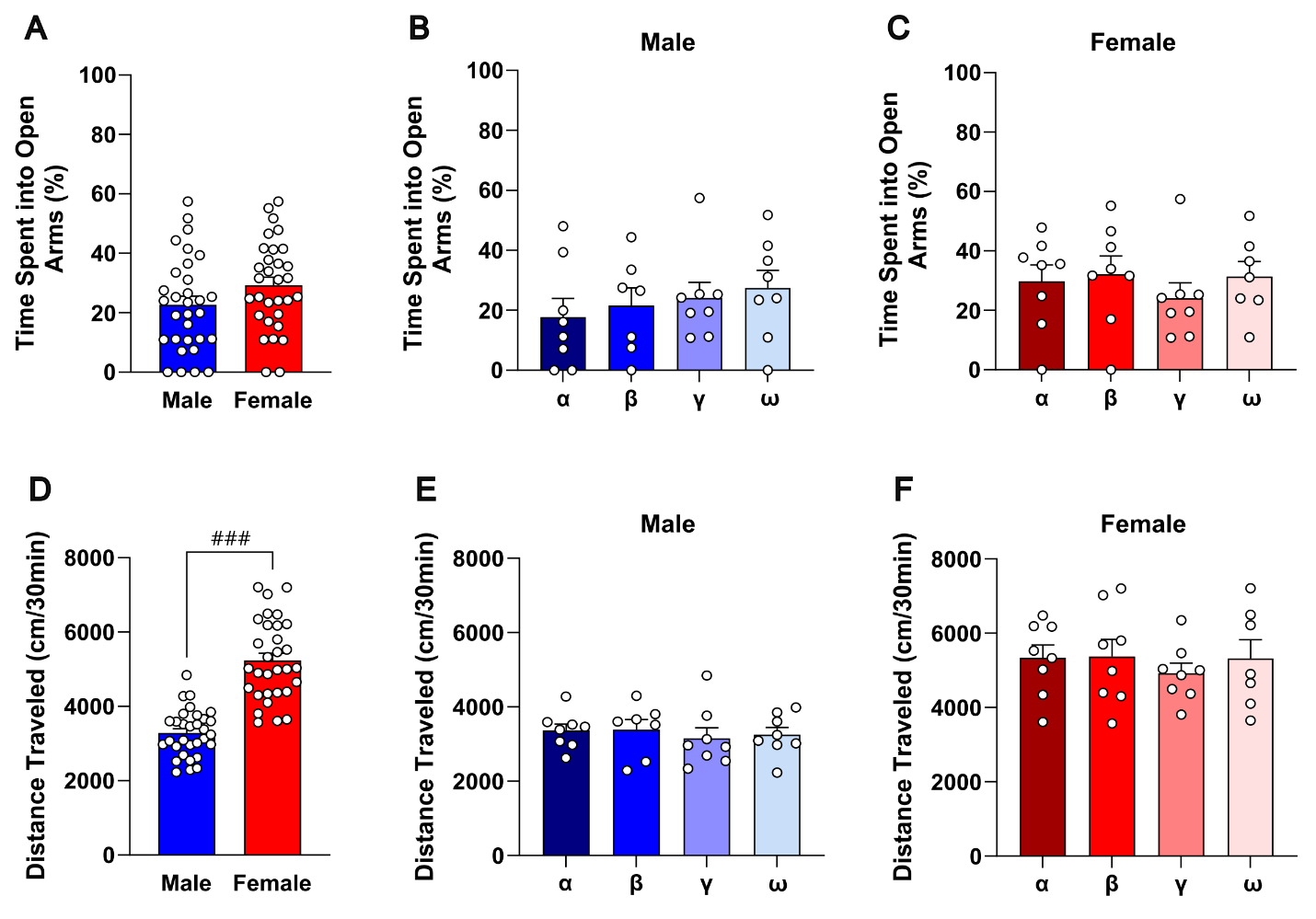
**

**Figure S7. A-B-C.** Time spent in the open arms during the Elevated Plus Maze in male and female rats (n = 31/sex) (**A**) and based on their social rank in males (**B**) and females (**C**). **D-E-F.** Distance traveled in a novel environment during the Open Field test in male and female rats (**D**) and based on their social rank in males (**E**) and females (**F**). Data are presented as mean ± SEM. ###*p* < 0.001 indicates a difference between sexes (male vs. female). Statistical analyses are reported in **Table S8**.

**
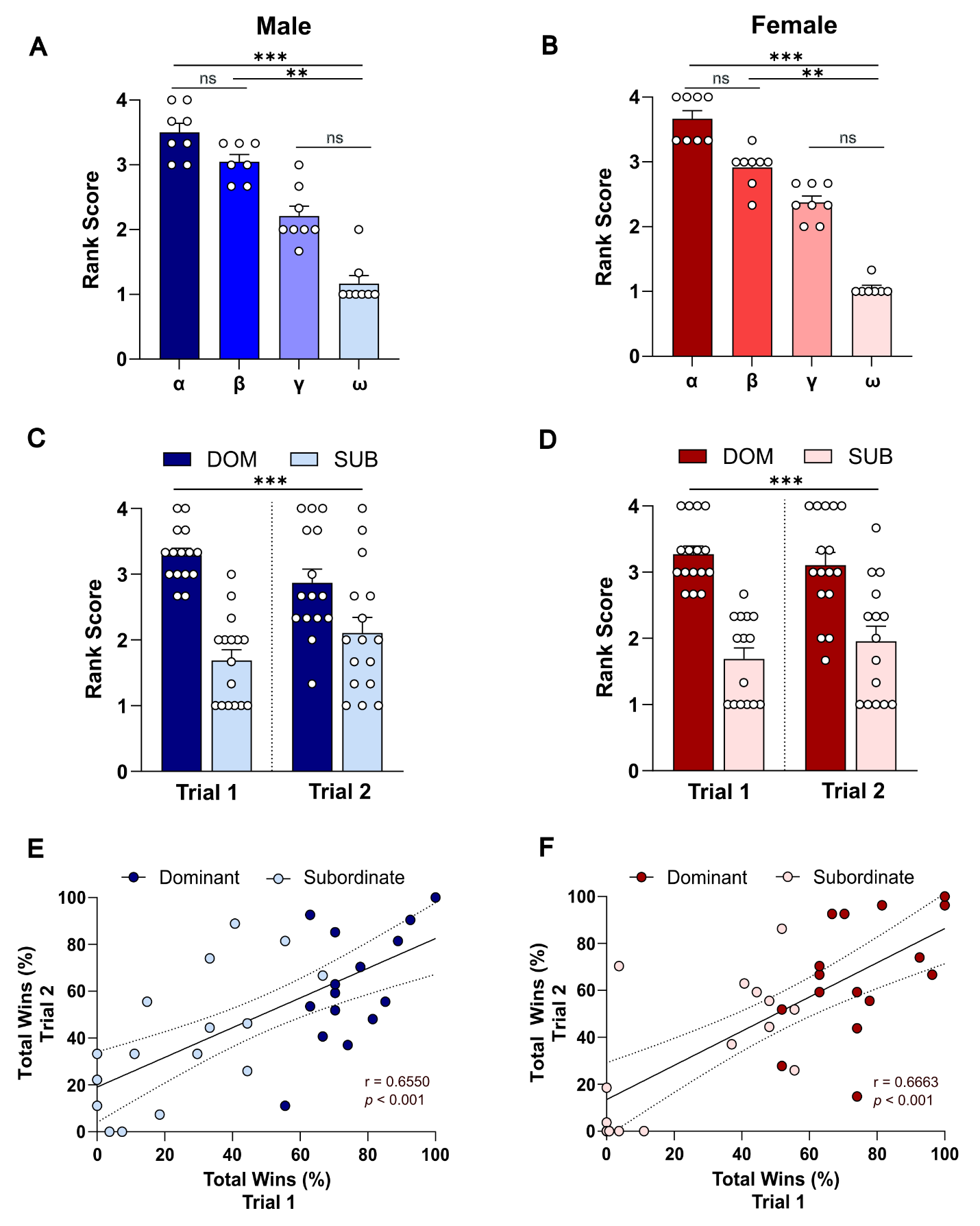
**

**Figure S8. A-B.** Total number of confrontations won during the Tube test in male (**A**) and female (**B**) rats (n = 31 per sex). **C-D.** Differences in rank scores between the first and second trials of the Tube test in male (**C**) and female (**D**) rats. **E-F.** Correlation between confrontations won during the first and second trials of the Tube test in male (**E**) and female (**F**) rats. Data are presented as mean ± SEM. ***p* < 0.01, ****p* < 0.001 indicate differences between social ranks (dominant vs. subordinate). Statistical analyses are reported in **Table S9**.

**
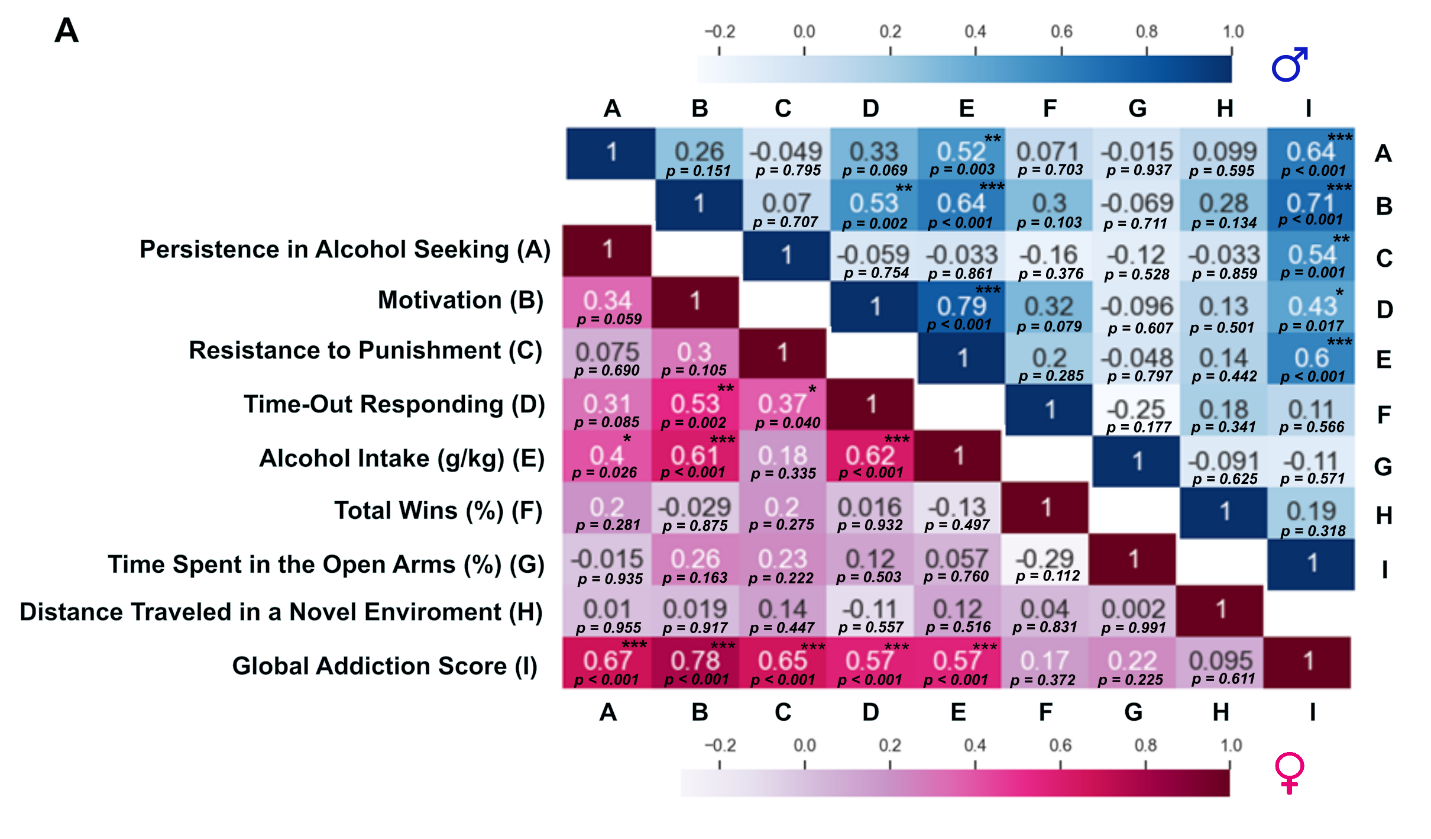
**

**Figure S9. A.** Correlation matrix of various behavioral measures in male and female rats (n = 31/sex) showed several correlations of interest. The global addiction score showed a strong correlation with alcohol intake in both males and females (for more details see **Fig. S10A-B**), suggesting that a greater history of alcohol consumption is linked to a higher likelihood of developing an AUD-like profile. Notably, in both sexes, neither anxiety-like and exploratory behaviors (EPM and OFT) nor social hierarchy correlated with the animals' global addiction score (for more details see **Fig. S10C-D**). Furthermore, alcohol intake did not correlate with resistance to punishment in either sex (for more details see **Fig. S10E-F**). Lastly, a strong correlation between the global addiction score and time-out responses was observed in both sexes.

**
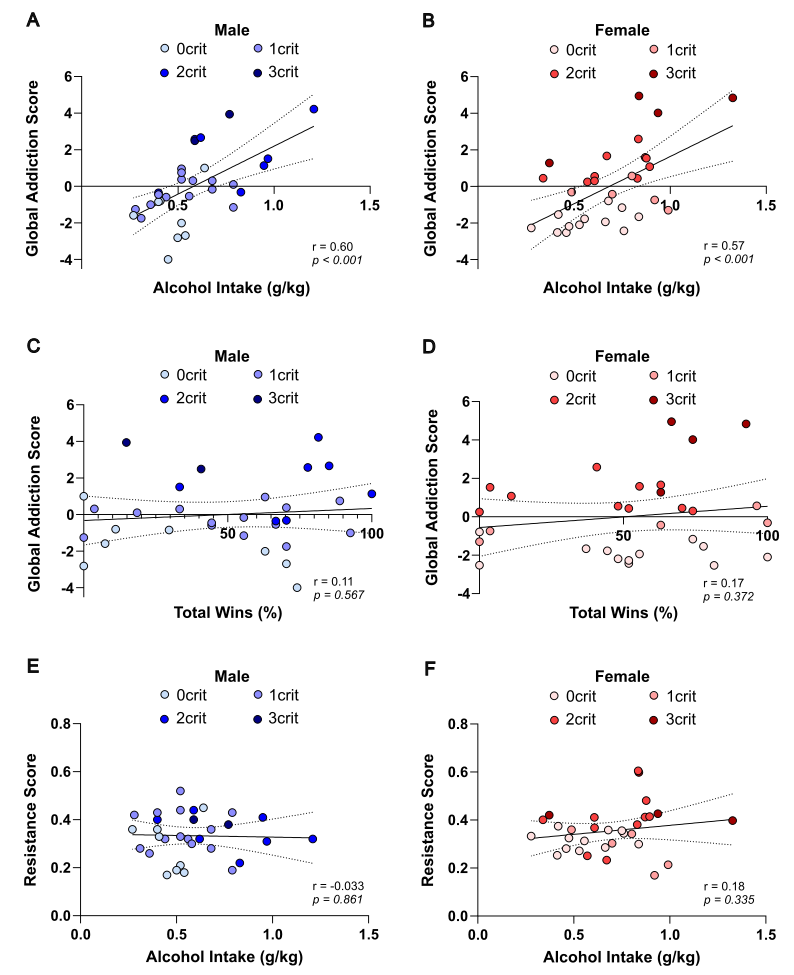
**

**Figure S10. A-B.** Correlation between the global addiction score and alcohol intake (g/kg) across the criteria groups in male (**A**) and female (**B**) rats (n=31/sex). **C-D.** Correlation between the global addiction score and total wins (%) in the tube test in male (**C**) and female (**D**) rats. **E-F.** Correlation between the resistance score and alcohol intake (g/kg) among the criteria groups in male (**E**) and female (**F**) rats.

**
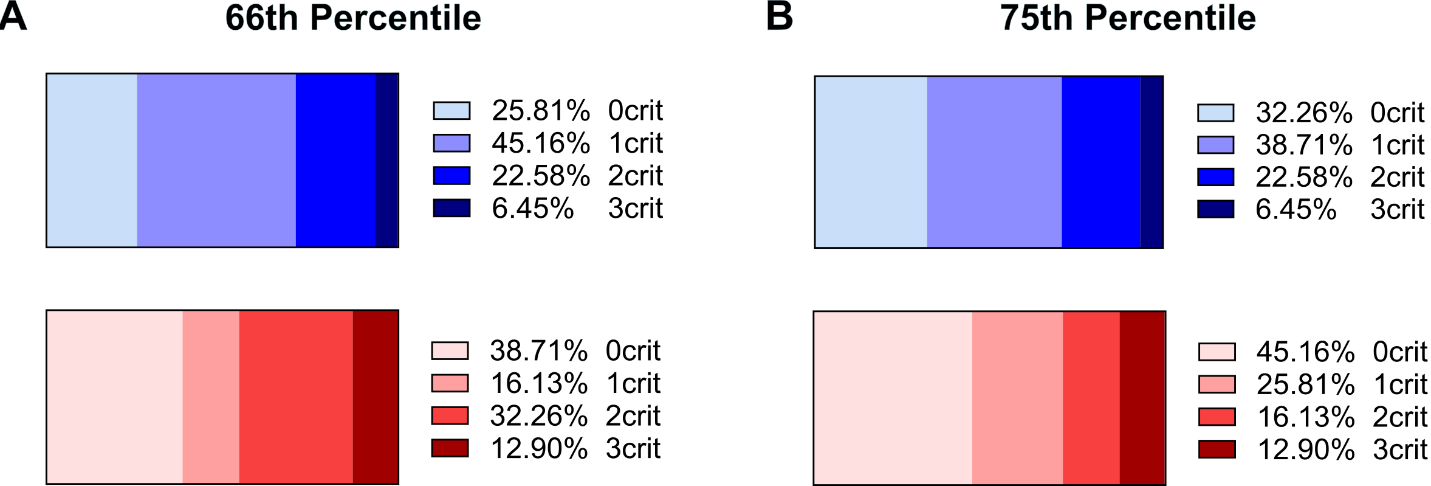
**

**Figure S11. Sex-specific distribution of addiction-like criteria varies depending on threshold used for classification.**

(**A**) and (**B**) show the percentage of animals classified as meeting 0, 1, 2, or all 3 addiction-like criteria (0crit–3crit) based on two different percentile thresholds for each criterion: the 66th percentile (**A**) and the 75th percentile (**B**). Upper panels: distribution in males. Lower panels: distribution in females. Statistical analyses are reported in **Table S11**.

**Table S1.** Summary of behavioral measures and session windows used to define each addiction-like criterion.

| **Addiction-like Criterion** | **Behavioral Measure** | **Session Window** |
| --- | --- | --- |
| **Persistence in Alcohol-Seeking** | Average number of active lever presses during no-drug periods | Sessions 37–60 (entire FR3 training phase) |
| **Motivation** | Breakpoint value during progressive ratio session | Single progressive ratio session |
| **Resistance to Punishment** | Average resistance score from five punished sessions | Punished sessions from the second punishment protocol |

**Table S2.** Statistical summary of PCA- and regression-based analyses exploring sex-dependent behavioral variability.

| **Number of figure** | **Statistic** | **Statistical details** |
| --- | --- | --- |
| **Figure S1B** | Two-Way ANOVA  ‘sex’ and ‘criteria’ as a between-subject factors | Sex: F_(1, 54)_ = 13.82, *p* < 0.001  Criteria: F_(3, 54)_ = 18.16, *p* < 0.001  Sex × Criteria: F_(3, 54)_ = 5.28, *p* < 0.01 |
| **Figure S1C** | Two-Way ANOVA  ‘sex’ and ‘criteria’ as a between-subject factors | Sex: F_(1, 54)_ = 43.37, *p* < 0.001  Criteria: F_(3, 54)_ = 5.40, *p* < 0.01  Sex × Criteria: F_(3, 54)_ = 0.75, *p* > 0.05 |
| **Figure S1D** | Multiple Linear Regression  The model included ‘sex’ and ‘criteria’ as main predictors, and the ‘sex × criteria’ interaction term to test whether the relationship between criteria and PC scores differed between sexes  (Dependent variable: PC1) | Model significance: p < 0.001  Sex: B = –0.302, SE = 0.439, β = –0.097, p = 0.494  Criteria: B = 1.406, SE = 0.233, β = 0.886, p < 0.001  Sex × Criteria: B = –0.709, SE = 0.296, β = –0.444, p = 0.020 |
| **Figure S1E** | Multiple Linear Regression  The model included ‘sex’ and ‘criteria’ as main predictors, and the ‘sex × criteria’ interaction term to test whether the relationship between criteria and PC scores differed between sexes (Dependent variable: PC2) | Model significance: p < 0.001  Sex: B = 1.449, SE = 0.321, β = 0.592, p < 0.001  Criteria: B = 0.364, SE = 0.171, β = 0.291, p = 0.037  Sex × Criteria: B = 0.140, SE = 0.217, β = 0.111, p = 0.522 |

**Table S3.** Statistical analysis of 20% alcohol self-administration training (active lever presses) during daily 30-minute sessions, divided into three 10-minute periods: P1 – alcohol available, P2 – alcohol not available, and P3 – alcohol available. Additionally, statistical analyses focusing exclusively on P2 (alcohol not available) are also reported.

| **Number of figure** | **Statistic** | **Statistical details** |
| --- | --- | --- |
| **Figure S2A** | Differences Across Periods (P1–P2–P3):  RM Two-Way ANOVA  ‘period’ as a between-subjects factor and ‘time’ as a within-subjects factor | Period: F_(2, 90)_ = 144.48, *p* < 0.001  Time: F_(59, 5310)_ = 74.05, *p* < 0.001  Period × time: F_(118, 5310)_ = 35.29, *p* < 0.001 |
| **Figure S2A** | Differences across days limited to P2:  RM One-Way ANOVA | F_(23, 690)_ = 1.509, *p* > 0.05 |
| **Figure S2B** | RM Two-Way ANOVA  ‘period’ as a between-subjects factor and ‘time’ as a within-subjects factor | Period: F_(2, 90)_ = 140.75, *p* < 0.001  Time: F_(59, 5310)_ = 41.31, *p* < 0.001  Period × time: F_(118, 5310)_ = 27.099, *p* < 0.001 |
| **Figure S2B** | Differences across days limited to P2:  RM One-Way ANOVA | F_(23, 690)_ = 1.52, *p* > 0.05 |

**Table S4.** Statistical analysis of time-out responding during 20% alcohol self-administration training.

| **Number of figure** | **Statistic** | **Statistical details** |
| --- | --- | --- |
| **Figure S3A** | RM Two-Way ANOVA  ‘sex’ as a between-subjects factor and ‘time’ as a within-subjects factor | Sex: F_(1, 60)_ = 11.997, *p* < 0.001  Time: F_(59, 3540)_ = 11.80, *p* < 0.001  Sex × time: F_(59, 3540)_ = 2.98 *p* < 0.001 |
| **Figure S3B** | RM Two-Way ANOVA  ‘sex’ as a between-subjects factor and ‘time’ as a within-subjects factor | Sex: F_(1, 60)_ = 15.64, *p* < 0.001  Time: F_(1, 60)_ = 23.198, *p* < 0.001  Sex × time: F_(1, 60)_ = 0.4955, *p* > 0.05 |
| **Figure S3C** | Generalized Linear Mixed Model  with robust covariance estimation | Criteria: F_(3, 54)_ = 0.166, *p* > 0.05  Time: F_(1, 54)_ = 2.033, *p* > 0.05  Criteria × time: F_(3, 54)_ = 0.722, *p* > 0.05 |
| **Figure S3D** | Generalized Linear Mixed Model  with robust covariance estimation | Criteria: F_(3, 54)_ = 0.908, *p* > 0.05  Time: F_(1, 54)_ = 7.154, *p* < 0.05  Criteria × time: F_(3, 54)_ = 0.847, *p* > 0.05 |

**Table S5.** Statistical analysis of resistance scores obtained during five punished alcohol self-administration sessions.

| **Number of figure** | **Statistic** | **Statistical details** |
| --- | --- | --- |
| **Figure S4A** | Generalized Linear Mixed Model | Criteria: F_(3, 135)_ = 1.898, *p* > 0.05  Time: F_(4, 135)_ = 2.325, *p* = 0.06  Criteria × time: F_(12, 135)_ = 0.618, *p* > 0.05 |
| **Figure S4B** | Generalized Linear Mixed Model | Criteria: F_(3, 135)_ = 7.500, *p* < 0.001  Time: F_(4, 135)_ = 1.729, *p* > 0.05  Criteria × time: F_(12, 135)_ = 0.760, *p* > 0.05 |

**Table S6.** Statistical analysis of resistance scores between male and female rats was conducted to compare their responses to the first shock received during the chained punishment session (10-minute) and their responses on the first day of the second punishment protocol (20-minute).

| **Number of figure** | **Statistic** | **Statistical details** |
| --- | --- | --- |
| **Figure S5** | RM Two-Way ANOVA  ‘sex’ as a between-subjects factor and ‘shock’ as a within-subjects factor | Sex: F_(1, 60)_ = 0.436, *p* > 0.05  Shock: F_(1, 60)_ = 183,6, *p* < 0.001  Sex × Shock: F_(1, 60)_ = 1.918, *p* > 0.05 |

**Table S7.** Statistical analysis of the number of rewards during the last three days of 20% alcohol self-administration training and differences in responses to footshock during the footshock sensitivity test.

| **Number of figure** | **Statistic** | **Statistical details** |
| --- | --- | --- |
| **Figure S6A** | Male: Kruskal-Wallis test  Female: One-Way ANOVA | H = 10.36, *p* < 0.05  F_(3, 27)_ = 2.999, *p* < 0.05 |
| **Figure S6B** | Male: Kruskal-Wallis test  Female: Kruskal-Wallis test | H = 4.393, *p* > 0.05  H = 2.625, *p* > 0.05 |

**Table S8.** Statistical analysis of time spent in the open arms during the Elevated Plus Maze test and distance traveled during the Open Field test.

| **Number of figure** | **Statistic** | **Statistical details** |
| --- | --- | --- |
| **Figure S7A** | Unpaired t-test | t = 1.678, df = 60, *p* < 0.05 |
| **Figure S7B** | One-Way ANOVA | F_(3, 27)_ = 0.509, *p* > 0.05 |
| **Figure S7C** | One-Way ANOVA | F_(3, 27)_ = 0.439, *p* > 0.05 |
| **Figure S7D** | Unpaired t-test | t = 8.747, df = 60, *p* < 0.001 |
| **Figure S7E** | One-Way ANOVA | F_(3, 27)_ = 0.214, *p* > 0.05 |
| **Figure S7F** | One-Way ANOVA | F_(3, 27)_ = 0.281, *p* > 0.05 |

**Table S9.** Statistical analysis of rank scores obtained during the different trials of the tube test.

| **Number of figure** | **Statistic** | **Statistical details** |
| --- | --- | --- |
| **Figure S8A** | Kruskal-Wallis test | H = 25.31, *p* < 0.001 |
| **Figure S8B** | Kruskal-Wallis test | H = 27.26, *p* < 0.001 |
| **Figure S8C** | Two-Way ANOVA  ‘social rank’ as a between-subjects factor and ‘trials’ as a within-subjects factor | Social rank: F_(1, 29)_ = 26.15, *p* < 0.001  Trials: F_(1, 29)_ = 0.00, *p* > 0.05  Social rank × Trials: F_(1, 29)_ = 9.883, *p* < 0.01 |
| **Figure S8D** | Two-Way ANOVA  ‘social rank’ as a between-subjects factor and ‘trials’ as a within-subjects factor | Social rank: F_(1, 29)_ = 39.16, *p* < 0.001  Trials: F_(1, 29)_ = 0.13, *p* > 0.05  Social rank × Trials: F_(1, 29)_ = 2.556, *p* > 0.05 |

**Table S10.** Statistical analysis of factor scores across animals grouped by the number of addiction-like criteria (0–3crit) in male and female rats.

| **Number of figure** | **Statistic** | **Statistical details** |
| --- | --- | --- |
| **Figure 4B – Factor 1** | Kruskal-Wallis test | H = 15.93, *p* < 0.01 |
| **Figure 4B – Factor 2** | Kruskal-Wallis test | H = 1.649, *p* > 0.05 |
| **Figure 4B – Factor 3** | Kruskal-Wallis test | H = 3.646, *p* > 0.05 |
| **Figure 4D – Factor 1** | One-Way ANOVA | F_(3, 27)_ = 9.96, *p* < 0.001 |
| **Figure 4D – Factor 2** | One-Way ANOVA | F_(3, 27)_ = 1.79, *p >* 0.05 |
| **Figure 4D – Factor 3** | One-Way ANOVA | F_(3, 27)_ = 3.01, *p <* 0.05 |

**Table S11.** Statistical analysis comparing the distribution of animals across the three criteria groups using the 66th and 75th percentile thresholds.

| **Number of figure** | **Statistic** | **Statistical details** |
| --- | --- | --- |
| **Figure S11A** | Chi-Square test | χ²(3) = 0.38, p = 0.94 |
| **Figure S11B** | Chi-Square test | χ²(3) = 2.51, p = 0.47 |

**REFERENCES**

1. Pellow S, Chopin P, File SE, Briley M. Validation of open:closed arm entries in an elevated plus-maze as a measure of anxiety in the rat. J Neurosci Methods. 1985;14(3):149-67.

2. Cao WY, Hu ZL, Xu Y, Zhang WJ, Huang FL, Qiao XQ, et al. Role of early environmental enrichment on the social dominance tube test at adulthood in the rat. Psychopharmacology (Berl). 2017;234(22):3321-34.

3. Jupp B, Murray JE, Jordan ER, Xia J, Fluharty M, Shrestha S, et al. Social dominance in rats: effects on cocaine self-administration, novelty reactivity and dopamine receptor binding and content in the striatum. Psychopharmacology (Berl). 2016;233(4):579-89.

4. Augier E, Dulman RS, Singley E, Heilig M. A Method for Evaluating the Reinforcing Properties of Ethanol in Rats without Water Deprivation, Saccharin Fading or Extended Access Training. J Vis Exp. 2017(119).

5. Augier E, Flanigan M, Dulman RS, Pincus A, Schank JR, Rice KC, et al. Wistar rats acquire and maintain self-administration of 20 % ethanol without water deprivation, saccharin/sucrose fading, or extended access training. Psychopharmacology (Berl). 2014;231(23):4561-8.

6. Garcia-Blanco A, Ramirez-Lopez A, Navarrete F, Garcia-Gutierrez MS, Manzanares J, Martin-Garcia E, et al. Role of CB2 cannabinoid receptor in the development of food addiction in male mice. Neurobiol Dis. 2023;179:106034.

7. Domi A, Cadeddu D, Lucente E, Gobbo F, Edvardsson C, Petrella M, et al. Pre- and postsynaptic signatures in the prelimbic cortex associated with "alcohol use disorder" in the rat. Neuropsychopharmacology. 2024;49(12):1851-60.

8. Hodos W. Progressive ratio as a measure of reward strength. Science. 1961;134(3483):943-4.

9. Domi A, Stopponi S, Domi E, Ciccocioppo R, Cannella N. Sub-dimensions of Alcohol Use Disorder in Alcohol Preferring and Non-preferring Rats, a Comparative Study. Front Behav Neurosci. 2019;13:3.

10. Pelloux Y, Everitt BJ, Dickinson A. Compulsive drug seeking by rats under punishment: effects of drug taking history. Psychopharmacology (Berl). 2007;194(1):127-37.

11. Hu Y, Salmeron BJ, Krasnova IN, Gu H, Lu H, Bonci A, et al. Compulsive drug use is associated with imbalance of orbitofrontal- and prelimbic-striatal circuits in punishment-resistant individuals. Proc Natl Acad Sci U S A. 2019;116(18):9066-71.

12. Goutaudier R, Joly F, Mallet D, Bartolomucci M, Guicherd D, Carcenac C, et al. Hypodopaminergic state of the nigrostriatal pathway drives compulsive alcohol use. Mol Psychiatry. 2023;28(1):463-74.

13. Seif T, Chang SJ, Simms JA, Gibb SL, Dadgar J, Chen BT, et al. Cortical activation of accumbens hyperpolarization-active NMDARs mediates aversion-resistant alcohol intake. Nat Neurosci. 2013;16(8):1094-100.

14. McDonald AJ, Nemat P, van 't Hullenaar T, Schetters D, van Mourik Y, Alonso-Lozares I, et al. Punishment-resistant alcohol intake is mediated by the nucleus accumbens shell in female rats. Neuropsychopharmacology. 2024;49(13):2022-31.

15. Marchant NJ, Campbell EJ, Kaganovsky K. Punishment of alcohol-reinforced responding in alcohol preferring P rats reveals a bimodal population: Implications for models of compulsive drug seeking. Prog Neuropsychopharmacol Biol Psychiatry. 2018;87(Pt A):68-77.

16. Bolles RC, Holtz R, Dunn T, Hill W. Comparisons of stimulus learning and response learning in a punishment situation. Learning and Motivation. 1980;11(1):78-96.

17. Deroche-Gamonet V, Belin D, Piazza PV. Evidence for addiction-like behavior in the rat. Science. 2004;305(5686):1014-7.

18. Belin D, Balado E, Piazza PV, Deroche-Gamonet V. Pattern of intake and drug craving predict the development of cocaine addiction-like behavior in rats. Biol Psychiatry. 2009;65(10):863-8.

19. Toivainen S, Xu L, Gobbo F, Della Valle A, Coppola A, Heilig M, et al. Different mechanisms underlie compulsive alcohol self-administration in male and female rats. Biol Sex Differ. 2024;15(1):17.

20. Domi E, Xu L, Toivainen S, Nordeman A, Gobbo F, Venniro M, et al. A neural substrate of compulsive alcohol use. Sci Adv. 2021;7(34).

21. Augier E, Barbier E, Dulman RS, Licheri V, Augier G, Domi E, et al. A molecular mechanism for choosing alcohol over an alternative reward. Science. 2018;360(6395):1321-6.

22. Virtanen P, Gommers R, Oliphant TE, Haberland M, Reddy T, Cournapeau D, et al. SciPy 1.0: fundamental algorithms for scientific computing in Python. Nat Methods. 2020;17(3):261-72.

23. Harris CR, Millman KJ, van der Walt SJ, Gommers R, Virtanen P, Cournapeau D, et al. Array programming with NumPy. Nature. 2020;585(7825):357-62.
